# Six weeks of strength endurance training decreases circulating senescence-prone T-lymphocytes in cytomegalovirus seropositive but not seronegative older women

**DOI:** 10.1186/s12979-019-0157-8

**Published:** 2019-07-25

**Authors:** Hung Cao Dinh, Ivan Bautmans, Ingo Beyer, Oscar Okwudiri Onyema, Keliane Liberman, Liza De Dobbeleer, Wim Renmans, Sam Vander Meeren, Kristin Jochmans, Andreas Delaere, Veerle Knoop, Rose Njemini

**Affiliations:** 1 Frailty in Ageing Research Group, Vrije Universiteit Brussel, Laarbeeklaan 103, B-1090 Brussels, Belgium; 2 Gerontology Department, Vrije Universiteit Brussel, Laarbeeklaan 103, B-1090 Brussels, Belgium; 3 Department of Geriatric Medicine, Universitair Ziekenhuis Brussel, Laarbeeklaan 101, B-1090 Brussels, Belgium; 4 Laboratory of Hematology, Universitair Ziekenhuis Brussel, Laarbeeklaan 101, B-1090 Brussels, Belgium; 5 Department of Internal Medicine, Pham Ngoc Thach University of Medicine, Ho Chi Minh City, Vietnam

**Keywords:** Community-dwelling older women, Cytomegalovirus infection, Immunosenescence, T-lymphocytes, Exercise, Strength endurance training

## Abstract

**Background:**

Ageing is associated with a decline in immune function termed immunosenescence. This process is characterized amongst others by less naive T-cells and more senescent phenotypes, which have been implicated in the pathogenesis of many age-related diseases. Thus far, reports regarding the long-term adaptation effects of exercise on T-cell phenotypes are scant and largely equivocal. These inconsistencies may be due to potential contributors to immunosenescence, particularly cytomegalovirus infection, which is considered a hallmark of T-cell senescence. Therefore, we sought to investigate the impact of cytomegalovirus serostatus on the distribution of peripheral T-cell subsets following long-term exercise in older women.

**Methods:**

One hundred women (aged 65 years and above) were randomized to 3 times/weekly training at either intensive strength training (3 × 10 repetitions at 80% of one-repetition maximum, *n* = 31), strength endurance training (2 × 30 repetitions at 40% of one-repetition maximum, *n* = 33), or control (passive stretching exercise, *n* = 36) for 6 weeks. All training sessions were supervised by trained instructors to minimize the risk of injury and to ensure that the participants adhered to the training protocol throughout the entire range of motion. The T-cell percentages and absolute blood counts were determined before and after 6 weeks (24 h–48 h after the last training session) using flow cytometry and a haematology analyser. Cytomegalovirus antibodies were measured in serum using Architect iSystem and cytomegalovirus serostatus was balanced in the three intervention groups. C-reactive protein was measured using immunonephelometry.

**Results:**

We report for the first time that 6 weeks of strength endurance training significantly decreased senescence-prone T-cells along with a small increase in the number of CD8– naive T-cells in blood. The absolute counts of senescent-like T-cells decreased by 44% (from 26.03 ± 35.27 to 14.66 ± 21.36 cells/μL, *p* < 0.01) and by 51% (from 6.55 ± 12.37 to 3.18 ± 6.83 cells/μL, *p* < 0.05) for the CD8+ and CD8– T-cell pools, respectively. Intriguingly, these changes were observed in cytomegalovirus seropositive, but not cytomegalovirus seronegative individuals.

**Conclusions:**

In conclusion, the present study shows that strength endurance training leads to a reduction in circulating senescence-prone T-cells in cytomegalovirus seropositive older women. It remains to be established if monitoring of peripheral senescence-prone T-cells may have utility as cellular biomarkers of immunosenescence.

**Electronic supplementary material:**

The online version of this article (10.1186/s12979-019-0157-8) contains supplementary material, which is available to authorized users.

## Introduction

Ageing is characterized by physiological changes, which are known to negatively impact different areas of the human body [1]. A severe change associated with this process is the progressive decline in immune response, referred to as immunosenescence. Of all the immune compartments, that of T-cells is among the most affected with ageing [2]. Advancing age leads to a shift in T-cell phenotypes, with a manifest decrease of naive T-cells - dealing with newly encountered antigens - and a concomitant accumulation of memory and senescent T-cells [3]. These changes are key contributors to the process of immunosenescence and are associated with greater risk of morbidity and mortality in older people [4]. Indeed, clinical evidence indicates that the ability to mount primary immune responses against novel antigens declines significantly with age [5], leading to the susceptibility of older people to often severe infectious diseases. Additionally, senescent T-cells are dysfunctional immune cells, which secrete increased amounts of pro-inflammatory substances and matrix degrading enzymes [6, 7], thereby creating a micro-environment favourable for the development of inflammatory diseases [8].

There are strong indications that infection with cytomegalovirus (CMV) may evoke the most deleterious effects on T-cell immunity due to the chronic antigenic load it delivers to T-cells [9, 10]. More so, evidence for frequent age-related reactivation and increased viral load of CMV in individuals with positive CMV serology has been reported [11, 12]. On the other hand, reactivation of the CMV infection has been postulated to trigger the immune response to generate highly oligoclonal T-cells. In this perspective, Khan et al. reported that age-related increase of memory CD8+ T-cells, is paralleled by an increase in the proportion of CMV epitope-specific T-cells [13]. In their study, individual CMV epitope-specific CD8+ T-cells could represent up to 23% of the total CD8+ T-cells in older adults with CMV infection. This clonal expansion of CMV-specific T-cells may exacerbate human T-cell immunosenescence and increase the susceptibility of older people to inflammatory processes [14].

Physical exercise is increasingly being recognized as a powerful countermeasure for immunosenescence and inflammation [15]. Although the mechanism underpinning exercise-induced immune response has not been completely elucidated, an overwhelming body of evidence, including some from our research group, suggests that regular bouts of exercise may improve immune function and lower inflammation [16–18] via the induction of anti-inflammatory cytokines and/or the inhibition of the expression of Toll-like receptors, with subsequent inhibition of the production of pro-inflammatory cytokines [19]. Another potential mechanism by which exercise may counteract immunosenescence and its associated diseases is by limiting the accumulation of senescent T-cells and repopulating blood with naive T-cells [20]. In this perspective, physical exercise has been shown to induce the mobilization of lymphocytes into the bloodstream, followed by the migration of lymphocytes to selected peripheral tissues for immune surveillance [21] with subsequent apoptosis of senescent T-cells [22]. This apoptotic process is thought to induce hematopoietic stem cell production in the bloodstream, which may move to the thymus and stimulate the development of naive T-cells [22].

Although cross-sectional studies indicate that regular exercise may combat the adverse effects of immunosenescence, available reports regarding the long-term adaptation benefits of exercise in the immune response of older persons are equivocal and most studies have either not examined or have failed to detect associations with CMV because almost all the older participants were CMV positive [23]. Considering the limited available data and ongoing controversies, we sought to study the effects of 6 weeks of resistance exercise at different intensities on circulating senescence-prone T-cells, with a particular focus on the role of CMV serostatus.

## Results

### Descriptive

The purpose of this study was to evaluate the role of CMV on the effects of 6 weeks resistance training at either intensive strength training (3 × 10 repetitions at 80% one-repetition maximum (1RM, i.e. the maximum weight that can be moved once over the whole range of movement)), strength endurance training (2 × 30 repetitions at 40% 1RM), or flexibility training (control) on blood T-cell subtypes in older women. The overall CMV seroprevalence was 65% and there were no differences in BMI or CRP levels between the CMV-seropositive and -seronegative groups. The participants with positive CMV serology were significantly older than the CMV seronegative participants (*p* = 0.029). Moreover, we found very weak, albeit significant, associations between age and the percentage of the memory phenotypes in both the CD8+ and CD8– T-cell subsets (r = 0.250, *p* = 0.012 and r = 0.243, *p* = 0.015; for CD8 + CD28 − CD57− and CD8 − CD28 − CD57−, respectively). Also, a significant increase was noticed in the proportion of CD8–CD57+ senescence-prone cells with age (r = 0.261, *p* = 0.009). On the other hand, a very weak inverse association was found between the proportion of naive cell phenotypes and age in both CD8+ and CD8– T-cell pools (r = − 0.329, *p* = 0.001 and r = − 0.243, *p* = 0.015; for CD8 + CD28 + CD57− and CD8 − CD28 + CD57−, respectively).

Overall muscle strength (muscle strength index representing the sum of 1RM on the 6 training devices) improved significantly after intensive strength training (+ 7.3 ± 4.5 kg, *p* < 0.01) and strength endurance training (+ 6.4 ± 4.5 kg, *p* < 0.01) compared to control (ANOVA post-hoc test, *p* < 0.001). Strength gains did not differ significantly between the intensive strength and strength endurance training groups (ANOVA post-hoc test, *p* = 0.485).

### Absolute counts and percentage of T-cells at baseline according to CMV serostatus

Table 1 portrays the baseline absolute counts and percentage of T-cell subsets according to CMV serostatus. In both CD8– and CD8+ T-cell pools, the seropositive CMV participants had higher absolute numbers of CD28–CD57– memory and CD28–CD57+ senescent phenotypes compared to their CMVseronegative counterparts (see Table 1). There were no significant differences in the absolute counts of CD8 − CD28 + CD57+ and CD8 + CD28 + CD57+ senescent phenotypes according to CMV serostatus. When age and CMV IgG were entered in a model together, we found that only CMV IgG was a significant predictor of both the number and percentage of senescence-prone T-cells (see Supporting Information Additional file 1: Tables S1 and Additional file 1: Table S2). There were no significant differences in the absolute counts of naive T-cells between the CMV-seropositive and -seronegative groups. Also, no significant differences in the absolute counts of T-cell phenotypes were noticed among the 3 intervention groups at baseline with respect to CMV serostatus (see Additional file 1: Table S3).

**Table 1:** Absolute counts and Percentage of T-cell subsets at baseline with respect to CMV serostatus

Parameter		CMV − (*n* = 34)	CMV + (*n* = 63)	*p* value^a^
T-cell subset				
CD8+ T-cells				
CD8 + CD28 + CD57− (naïve)	cells/μL	130.29 (88.50)	154.60 (117.30)	0.111
	%	73.60 (24.40)	54.90 (26.40)	< 0.001
CD8 + CD28 − CD57− (memory)	cells/μL	30.00 (52.50)	90.00 (100)	< 0.001
	%	20.30 (18.53)	29.50 (22.30)	< 0.001
CD8 + CD57+ (SPC)	cells/μL	5.81 (17.30)	27.06 (38.30)	< 0.001
	%	4.00 (7.15)	7.15 (13.08)	0.010
CD8 + CD28 − CD57+ (SPC)	cells/μL	4.90 (15.70)	26.38 (37.40)	< 0.001
	%	2.90 (6.25)	6.35 (11.98)	0.004
CD8 + CD28 + CD57+ (SPC)	cells/μL	0.84 (1.40)	0.65 (1.40)	0.913
	%	0.30 (0.80)	0.20 (0.40)	0.065
CD8− T-cells				
CD8 − CD28 + CD57 − (naive)	cells/μL	641.91 (310.20)	650.27 (343.30)	0.844
	%	99.40 (0.90)	96.50 (4.70)	< 0.001
CD8 − CD28 − CD57− (memory)	cells/μL	0.00 (10.00)	20.00 (20.00)	< 0.001
	%	0.45 (0.65)	2.00 (4.00)	< 0.001
CD8 − CD57+ (SPC)	cells/μL	0.66 (1.50)	6.92 (13.80)	< 0.001
	%	0.10 (0.20)	0.90 (2.00)	< 0.001
CD8 − CD28 − CD57 + (SPC)	cells/μL	0.00 (0.80)	6.12 (10.00)	< 0.001
	%	0.00 (0.13)	0.80 (1.70)	< 0.001
CD8 − CD28 + CD57 + (SPC)	cells/μL	0.56 (0.90)	0.81 (1.80)	0.081
	%	0.10 (0.10)	0.10 (0.40)	0.151

Note: The values denote median (Interquartile range). *CMV* cytomegalovirus, *SPC* senescence-prone cells. T-cell subsets were expressed as percentages within the CD3 + CD8+ or CD3 + CD8− T-cells or absolute number of cells in peripheral blood (cells/μL). ^a^Results of Mann-Whitney U Test

Regarding the baseline proportion of cells, the CMV seropositive group was characterized by a significantly lower proportion of the naive phenotype - defined as CD28 + CD57– expressing cells - in both the CD8+ and the CD8– sub-populations of T-cells (all *p* < 0.001, see Table 1). Contrariwise, the proportions of CD28–CD57–memory cells and CD28–CD57+ senescent phenotypes were significantly lower in participants without CMV compared to CMV–seropositive participants, in both lineage markers of the lymphocyte subset (all *p* < 0.05, see Table 1). There were no significant differences in the percentage of CD8 − CD28 + CD57+ or CD8+ CD28 + CD57+ senescent phenotypes according to CMV serostatus. Also, no significant differences in the percentage of T-cell phenotypes were noticed among the 3 intervention groups at baseline with respect to CMV serostatus (see Additional file 1: Table S3).

### Training-induced changes in the absolute counts of T-cell subsets

Considering the huge impact of CMV on the absolute counts of various T-cell subsets, we analysed the absolute blood counts of the various T-cell subsets stratifying by CMV serostatus.

### CD8+ T-cell subset

**Fig. 1:**
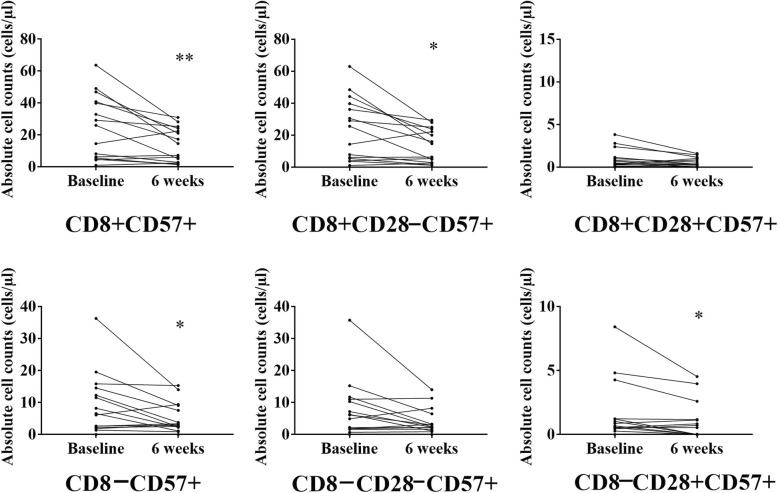
Training-induced changes in the absolute counts of senescent phenotypes in the strength-endurance training intervention group in CMV seropositive participants. Note: The points represent experimental data for 15 individuals. Results of Wilcoxon signed-rank test for pooled data from all the samples; asterisk (∗) (*p* < 0.05) and double asterisks (∗∗) (*p* < 0.01) indicate significant decrease after exercise compared to baseline

For the entire CMV seropositive group, we found a significant increase in the absolute number of CD8 + CD28–CD57– memory cells (*p* = 0.025, see Additional file 2: Figure S1). When CMV+ participants were categorized according to intervention group, within group analysis revealed an exercise-induced decrease in the absolute number of the senescent phenotypes (CD8 + CD57+ and CD8 + CD28 − CD57+) in the strength endurance training intervention group (all *p* < 0.05, see Fig. 1). Further, pairwise group comparisons revealed a significant decrease in the training-induced changes for the absolute counts of CD8 + CD57+ and CD8 + CD28 − CD57+ in the strength endurance training intervention group compared to the control group (*p* = 0.050, and *p* = 0.036, respectively).

### CD8– T-cell subset

For the CD8– T-cell subset, we found a very small, albeit significant, increase in the absolute number of the CD8–CD28 + CD57– naive phenotype in the CMV+ participants (*p* = 0.047, see Additional file 2: Figure S1 and Additional file 1: Table S4). Also, there was a significant decrease in the CD8–CD57+ senescent phenotype following training compared to pre-training levels in CMV seropositive participants (*p* = 0.037, see Additional file 1: Table S4). When CMV+ participants were categorized according to intervention group, within group analysis revealed an exercise-induced decrease in the absolute number of the senescent phenotypes (CD8 − CD57+ and CD8 − CD28 + CD57+) only in the strength endurance training intervention group (all *p* < 0.05, see Fig. 1).

**Fig. 2:**
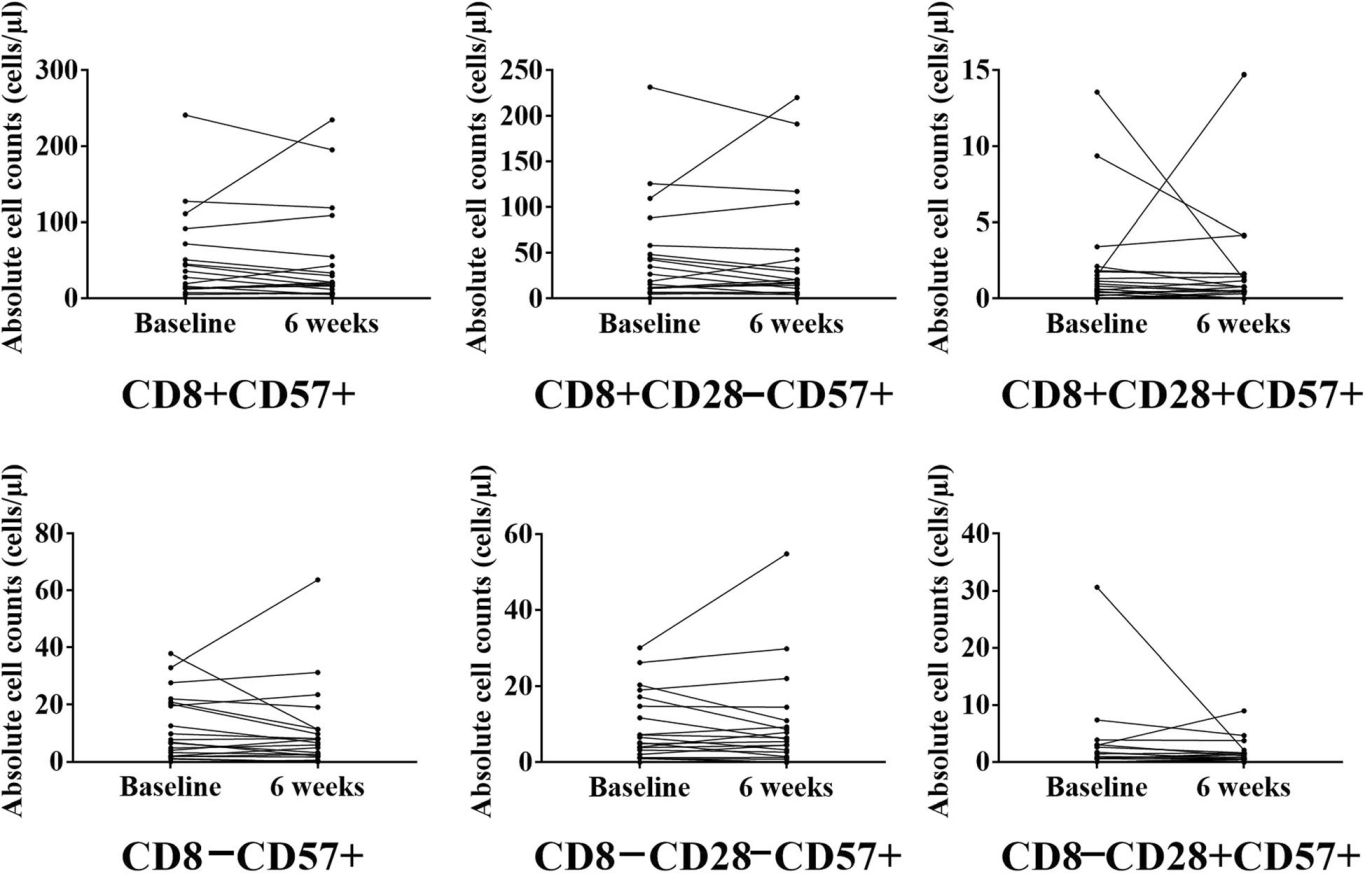
Training-induced changes in the absolute counts of senescent phenotypes in the intensive strength training intervention group in CMV seropositive participants, Note: The points represent experimental data for 21 individuals

**Fig. 3:**
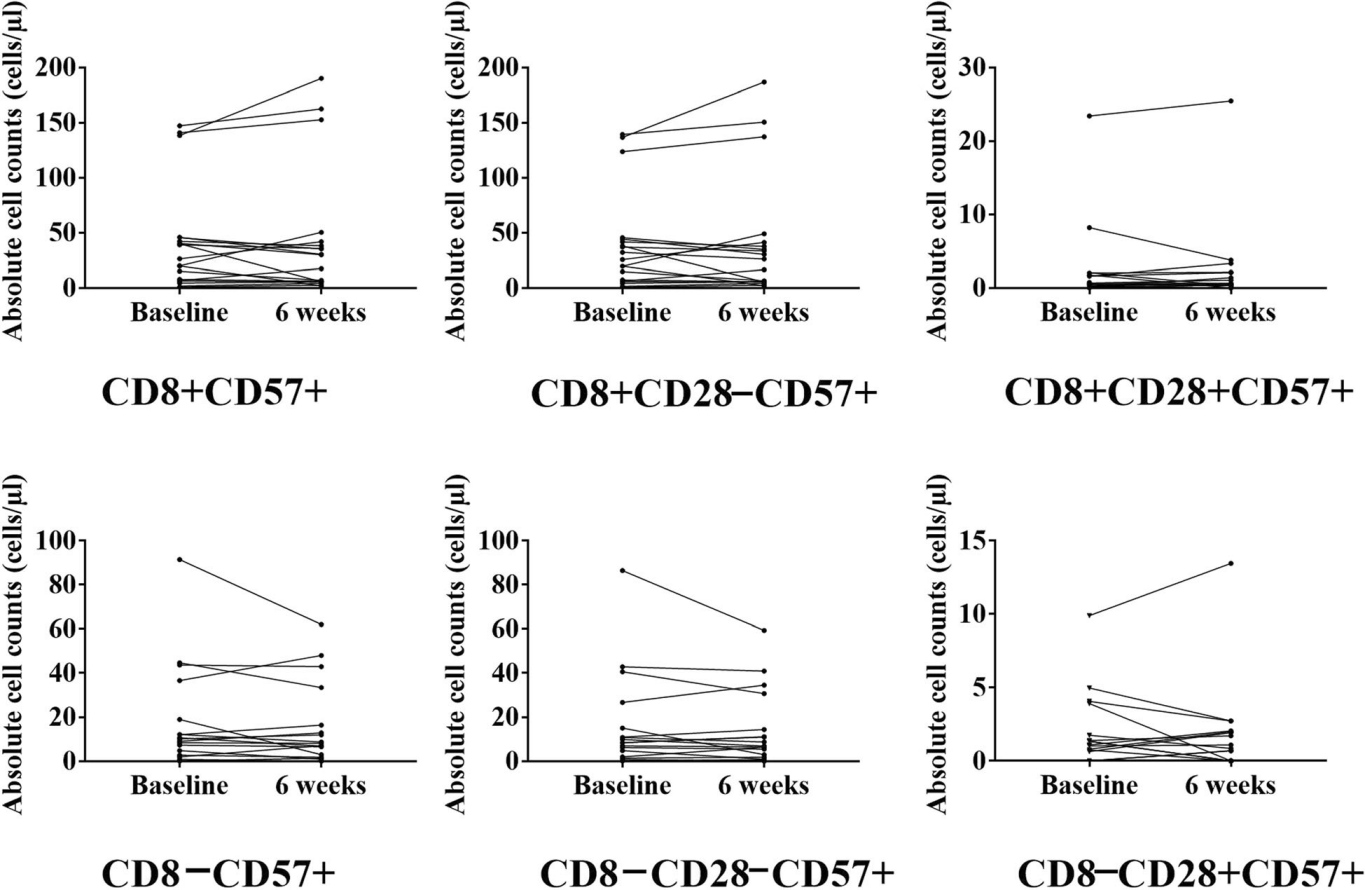
Training-induced changes in the absolute counts of senescent phenotypes in the control group in CMV seropositive participants. Note: The points represent experimental data for 19 individuals

We did not find any differences in the absolute counts of T-cell subsets after intensive strength training or flexibility training (see Figs. 2 and 3, respectively). Also, we did not find any differences in the absolute counts of T-cell subsets after exercise compared to pre-exercise counts within the CMVseronegative participants (see Additional file 1: Table S5 and Additional file 2: Figure S1).

### Training-induced changes in the proportion of T-cell subsets

To understand if the exercise-induced changes in the absolute counts of T-cell subsets are reflected in their proportion in peripheral blood, we analysed pre-post training changes in the proportion of T-cell phenotypes in CMV seropositive and CMV seronegative participants separately (see Table 2 and Additional file 1: Table S6).

**Table 2:** Training-induced changes in the percentage of T-cell subsets among the different intervention groups in CMV seropositive participants

T-cell subset	IST (*n* = 21)	SET (*n* = 16)	CON (*n* = 20)	Time effect^a^	Time* group effect^b^
CD8+ T-cells					
CD8 + CD28 + CD57− (naive)					
CD8 − CD57+ (SPC)Baseline	50.50 (27.75)	60.75 (23.68)	50.85 (31.53)	0.357	0.296
6 weeks	48.50 (33.70)*	62.55 (26.47)	52.25 (27.63)		
CD8 + CD28− CD57− (memory)					
Baseline	28.00 (22.55)	33.10 (27.25)	32.00 (23.17)	0.039	0.940
6 weeks	37.30 (25.70)	30.45 (27.50)	33.45 (23.75)		
CD8 + CD57+ (SPC)					
Baseline	5.60 (17.13)	6.25 (11.55)	7.45 (12.38)	0.012	0.161
6 weeks	6.70 (10.48)	5.00 (6.50)**	3.55 (15.63)		
CD8 + CD28 − CD57+ (SPC)					
Baseline	5.45 (16.50)	6.15 (10.83)	6.65 (11.73)	0.011	0.128
6 weeks	6.20 (10.03)	4.65 (6.48)**	3.30 (15.20)		
CD8 + CD28 + CD57+ (SPC)					
Baseline	0.25 (0.48)	0.10 (0.38)	0.20 (0.48)	0.249	0.673
6 weeks	0.20 (0.50)	0.10 (0.43)	0.15 (0.48)		
CD8− T-cells					
CD8 − CD28 + CD57− (naive)					
Baseline	96.40 (5.35)	96.55 (5.78)	95.60 (5.45)	0.727	0.307
6 weeks	96.90 (5.35)	97.20 (5.47)	96.55 (7.17)		
CD8 − CD28− CD57− (memory)					
Baseline	1.80 (4.80)	2.30 (4.50)	2.75 (3.70)	0.091	0.701
6 weeks	2.10 (3.75)	1.85 (4.33)	2.55 (2.68)		
CD8 − CD57+ (SPC)					
Baseline	1.10 (2.60)	0.75 (1.85)	1.00 (2.63)	0.027	0.331
6 weeks	1.00 (1.80)	0.40 (0.65)*	0.80 (1.85)		
CD8 − CD28 − CD57+ (SPC)					
Baseline	1.00 (1.60)	0.70 (1.68)	0.90 (2.10)	0.034	0.337
6 weeks	0.80 (1.75)	0.40 (0.48)*	0.75 (1.55)		
CD8 − CD28 + CD57+ (SPC)					
Baseline	0.10 (0.40)	0.10 (0.08)	0.10 (0.35)	0.152	0.238
6 weeks	1.00 (0.15)	0.05 (0.10)*	0.10 (0.28)		

Note: The values denote median (Interquartile range). *CMV* cytomegalovirus, *SPC* senescence-prone T-cells, *IST* intensive strength training, *SET* strength-endurance training, *CON* control. T-cell subsets were expressed as percentages within the CD3 + CD8+ or CD3 + CD8− T-cells. ^a^Results of Wilcoxon signed-rank test for changes between baseline and 6 weeks for the entire CMV+ cohort; that is IST, SET and CON combined. ^b^Results of Kruskal-Wallis test for changes between baseline and 6 weeks (a real numerical value was computed for each individual) among the 3 groups of training. Wilcoxon’s signed-rank test for within group differences: **p < 0.01 and *p < 0.05 significantly different from baseline within group

### CD8+ T-cell subset

In the CMV seropositive population, we found a significant decrease in the proportion of CD8+ senescent cells (CD8 + CD57+, *p* = 0.012) - likely due to the CD8 + CD28–CD57+ variant (*p* = 0.011) as CD8 + CD28 + CD57+ did not change significantly - and an increase in the CD8 + CD28–CD57– highly differentiated memory phenotype (*p* = 0.039) after 6 weeks training compared to baseline (see Table 2). Within group analysis revealed a significant decrease in the proportions of the senescent phenotypes (CD8 + CD57+ and CD8 + CD28–CD57+) after training only in the strength endurance training intervention group (all *p* < 0.01, see Table 2). Pairwise group comparisons revealed a significant decrease in the training-induced changes for CD8 + CD57+ and CD8 + CD28 − CD57+ in the strength endurance training intervention group compared to the control group (*p* = 0.047, and *p* = 0.042, respectively).

### CD8– T-cell subset

In the CD8– T-cell compartment, we found a significant decrease in the CD8–CD57+ senescent cells (*p* = 0.027, mostly due to the CD8–CD28–CD57+ subpopulation (*p* = 0.034)). Within group analysis showed a significant decrease in the proportions of the senescent phenotypes (CD8 − CD57+, CD8 − CD28 − CD57+, and CD8 − CD28 + CD57+) after training only in the strength endurance training intervention group (all *p* < 0.05, see Table 2). Moreover, a weak inverse correlation was found between the exercise-induced changes in the proportion of CD8–CD28 + CD57– naive phenotype and the decline in the proportion of CD8–CD57+ (r = − 0.443, *p* < 0.01), CD8–CD28–CD57+ (r = − 0.400, *p* < 0.01), and CD8–CD28 + CD57+ (r = − 0.370, *p* < 0.01) senescent subsets in the CMV+ cohort post-training. No such correlations were seen in the CD8+ T-cell pool.

No significant time by group interaction effects were noticed in the percentage of T-cell phenotypes from baseline to post exercise in the CMV seropositive group (see Table 2). Regarding the CMV seronegative group, no significant exercise-induced changes were recorded in the proportions of T-cell phenotypes (see Additional file 1: Table S6).

## Discussion

Findings from cross-sectional studies indicate that regular exercise may limit the accumulation of senescent cells and counter the adverse effects of immunosenescence. However, evidence pertaining to the long-term adaptation benefits of exercise in older people is equivocal and less convincing. Although this might indicate that long-term training exerts little restoration properties [24], it is probable that variation in experimental design and the health status of participants influence immunological responses to physical activity [25]. In view of our previous research showing a preferential accumulation of senescence-prone cells in CMV seropositive participants [26] and because exercise induces changes in T-cell subset distribution, we investigated the impact of CMV serostatus on T-cell response to different exercise intensities in older women. Our results show that strength endurance training leads to a reduction in circulating senescence-prone T-cells in CMV seropositive elderly women. The functional consequences for immunosenescence remain to be investigated.

We found that 6 weeks of strength endurance training significantly lowered senescence-prone T-cells in CMV seropositive, but not seronegative, older women. This could be attributed, at least in part, to exercise-induced changes in T-cell subset distribution. Acute exercise has been shown to induce the influx of lymphocytes - preferentially the late stage differentiated senescent-like phenotypes [27] - into the bloodstream, and their subsequent migration to selected peripheral tissues, with consequent apoptosis of a portion of the senescent T-cells [21]. This exercise dependent mobilization of cells is driven in part by increased shear forces and blood pressure during exercise [28], but most importantly through stimulation of beta-2-adrenergic receptors on the surface of lymphocytes by hormones released during exercise [29]. Given that CMV specific T-cells express higher levels of β-adrenergic receptors [9], it can be expected that exercise would induce a more efficient influx of CMV specific T-cells into the peripheral circulation compared to the broader T-cell pool. In this light, scant reports have investigated the influence of CMV status on exercise-induced changes in T-cell subset distribution [30], with results indicating a higher exercise-induced redeployment of highly differentiated T-lymphocytes - mostly specific for CMV - among the CMV positive participants. Therefore, frequent exercise-induced mobilization of CMV-specific T-cells - with subsequent apoptosis of some of them in the peripheral tissues - may produce a cumulative reduction in the number of cells with senescent phenotype over time in participants with CMV.

Another obvious potential mechanism by which exercise may diminish circulating senescence-prone cells in participants infected with CMV is by lowering inflammation. Regular exercise training has been unequivocally reported to reduce circulating resting levels of the pro-inflammatory cytokines IL-6 and TNF-α in older people [31]. In response to 24 weeks of resistance exercise training, we also observed a significant decrease in the resting levels of IL-6 in community dwelling older individuals [32]. On the other hand, chronic low-grade inflammation has been proposed to perpetuate the reactivation of CMV [33]. Because the reactivation of CMV infection has been postulated to trigger the immune response to generate highly oligoclonal T-cells - which may become senescent over time [34] - reducing inflammation via exercise may limit the opportunities for latent viral agents to reactivate and thereby reduce the accumulation of senescent cells in participants presenting with CMV.

Finally, our observation could be due to the stress-reducing properties of strength endurance training. Vincent et al. reported that older people who underwent training at a lower intensity with more repetitions (50% 1RM; 13 repetitions per exercise) experienced a more pronounced protective effect against lipid peroxidation than their counterparts who trained at a higher intensity with fewer repetitions (80% 1RM; 8 repetitions per exercise) [35]. Moreover, oxidative stress is known to trigger the reactivation of latent CMV infection [36], which could lead to the development of senescent phenotypes in the T-cell pool. Thus, strength endurance training may decrease the number of senescence-prone T-cells in CMV positive participants perhaps by lowering oxidative stress. Taken together, strength endurance training may delay the accumulation of senescence-prone cells in seropositive CMV individuals directly by inducing the apoptosis of senescence-prone cells, or indirectly by limiting inflammation and oxidative stress, both of which drive viral reactivation.

In the present study, we found a significant increase in the proportion of memory cells after 6 weeks intervention in CMV seropositive participants. In accordance with this finding, Silva et al. reported a higher proportion of highly differentiated effector memory cells in CMV seropositive elderly individuals with an exercise lifestyle, and they concluded that such effects may be beneficial for the immune response to known antigens, such as pathogens specific to CMV [37]. Regarding the naive T-cell pool, we found a significantly lower proportion of the CD8 + CD28 + CD57– naive T-cells in CMV+ compared to CMV– participants at baseline. On the other hand, a higher, albeit not significant, number of CD8 + CD28 + CD57– naive T-cells was observed in CMV+ compared to CMV– participants at baseline. The relative lower percentage of naive CD8+ cells in CMV+ as opposed to CMV– may be explained by the higher absolute numbers of memory and senescent T-cell subsets in the CD8+ compartment in CMV+ participants. In the CD8− compartment, we observed a very small, albeit significant, training-induced increase in the absolute counts of naive T-cells among CMV positive participants. More so, we found a weak inverse correlation between the training-induced changes in the proportion of CD8 − CD28 + CD57− naive T-cells and the decline in the proportion of CD8 − CD57+, CD8 − CD28 − CD57+, and CD8 − CD28 + CD57+ senescence-prone phenotypes post-training. However, these results should be interpreted with caution since the training-induced changes in the absolute counts of CD8 − CD28 + CD57– naive T-cells did not correlate significantly with the decline in absolute counts of CD8− senescent T-cells. Moreover, we did not find any significant changes in CD8 + CD28 + CD57– naive T-cell subset following training and no significant relationships were found between the training-induced changes in CD8 + CD28 + CD57– naive T-cells and the decline in CD8+ senescent phenotypes post-training.

As expected, our findings support the current knowledge that T-cell repertoire are altered - resulting in a decrease of naive cells, with a concomitant accumulation of memory and senescent cells - during ageing. This age-dependent shift from T-cells exhibiting predominantly naive phenotypes to highly differentiated memory and senescent phenotypes might reflect lower numbers of haematopoietic stem cells, and thymus involution with age [38]. Notwithstanding, when age and CMV IgG were entered in a model together, we found that only CMV IgG was a significant predictor of both the number and percentage of senescence-prone T-cells.

Although the overall muscle strength improved significantly after both intensive strength training and strength endurance training, 6 weeks of intensive strength training did not influence significantly the blood counts of senescence-prone T-cells in CMV-seropositive or -seronegative older women. Since reports addressing the immune response to resistance training in older individuals are lacking, more functional studies are highly needed to understand the significance of our observation in the context of immunosenescence. Certainly, more research is required to elucidate the possible role played by the chemokines, cytokines, oxidative stress, gender, lifestyle variables and infection history on the distribution of T-cell phenotypes following exercise.

The strengths of this study are many folds. A strong point is that this study was performed in an older population with a distinctly different physiologic profile compared to younger subjects. Thus far, the scant data in this field have not addressed the impact of CMV on the long-term adaption effects of exercise on T-cells. Therefore, this study addresses a gap in the literature by investigating the impact of CMV on exercise-induced T-cell adaptation in older women. Further, the available data in this field have largely used the classical aerobic training. Research pertaining to resistance training - which is very well suited to older people’s diverse circumstances - has been largely unexploited in the context of the ageing immune system. Our observation that long-term strength endurance training modulates T-cell phenotypes in CMV seropositive, but not seronegative individuals, will act as a guide for future experiments concerning T-cell response to exercise in older people. Although the present study has a promising setup, the findings should be interpreted within its limitations. First, T-cell subsets were distinguished based on the expression (or non-expression) of CD28 and CD57. However, the expression of CD28 by CD8+ and CD8– T-cells indicates different stages in lymphocyte differentiation as memory CD8+ T-cells tend to lose CD28 expression before losing CD27, while the reverse is true for CD4+ T-cells [39]. Therefore, additional T-cell markers (e.g. CD45RA, CCR7, CD4 and CD27) might be useful to better define different T-cell subsets and their response to exercise. A second limitation is the lack of a young control or male group, which might have provided insight into the respective effects of ageing and sex on exercise-induced adaptations of immune cells. Third, we did not determine the CMV specific T-cells. Therefore, we could not verify the share of CMV-specific T-cells in mobilized T-cells in response to strength endurance training. Clearly, more research is needed in this area that employs a more robust measurement of immune competency including immune cell phenotypes and their functionality (e.g. their capacity to proliferate, and to produce inflammatory substances), as well as lifestyle measurements (e.g. level of physical activity, nutrition and body composition) while keeping in mind individuals’ immune profiles particularly CMV status (e.g. the IgM class anti-CMV antibodies, PCR assay for CMV and genetic predisposition to CMV infection). Since reports addressing the immune response to resistance training in older individuals are lacking, more functional studies are highly needed to understand the significance of our observation in the context of immunosenescence. This could be done for instance by assessing the effects of SET on vaccine responses. Also, questions as to whether senescence-prone cells die or track to the marginal pool or tissues should be addressed in future studies.

## Conclusion

In conclusion, the results of the present study show that strength endurance training leads to a reduction in circulating senescence-prone T-cells in seropositive CMV older women. It remains to be established if monitoring of peripheral senescence-prone T-cells may have utility as cellular biomarkers of immunosenescence.

## Methods

### Participants and study design

**Fig. 4:**
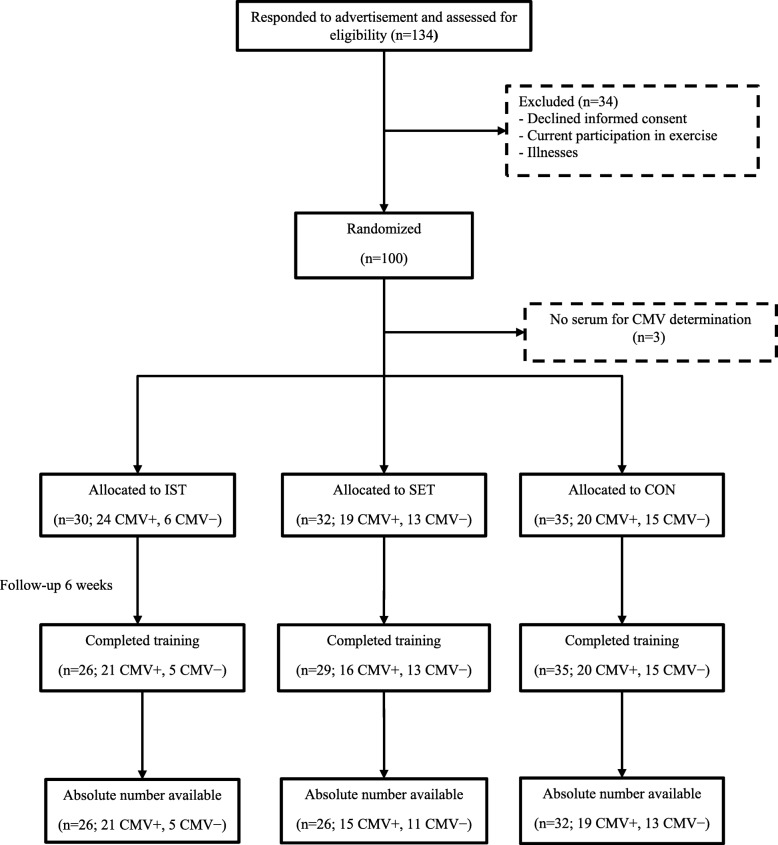
Flowchart of study participants. Note: CMV = cytomegalovirus; IST = intensive strength training; SET = strength endurance training; CON = control

The Senior Project Intensive Training (SPRINT) is an ongoing randomized controlled trial conducted by the Frailty in Ageing research department of Vrije Universiteit Brussel to evaluate the effects of resistance training at different intensities on the immune system in elderly persons. Recruitment was done by advertisement with flyers through day centres, health insurance companies, seniors associations, general practitioners, municipalities and other public places. Participants were excluded when performing currently or within the past 6 months, on a regular basis, physical exercise at higher intensities than habitual daily activity (e.g. fitness classes, strengthening exercises, cycling club); when presenting contra-indication for any of the exercise interventions; when using corticosteroids; when being unable to understand or execute the exercise instructions due to cognitive impairment (mini mental state examination score < 24/30) [40] or physical disability. Comorbidity was not an exclusion criterion per se, except for acute uncontrolled conditions and/or acute inflammation (C-reactive protein (CRP) ≥ 10 mg/L). One hundred apparently healthy older women (aged 65 years and above) living independently in the community were included in the present study (see Fig. 4). Among those randomized, 3 had no serum for CMV determination, 7 were lost to follow up and the results of absolute counts of T-cells could not be retrieved for 6 participants. The study protocol was approved by the local ethics committee in accordance with the Declaration of Helsinki and each participant gave a written informed consent.

### Health categories and randomization

Participants were initially classified into health categories (see Table 3) based on a modified SENIEUR’s protocol and according to the risk for complications during physical training as described previously [32, 42, 43]. Thereafter, they were randomly assigned to 6 weeks of either intensive strength training, strength-endurance training, or control (see Fig. 4). Randomization was stratified for age (65–74 / ≥75 years) and health status (see Table 3). Group allocation was performed by a researcher who was blinded for study outcomes and allocation sequence.

**Table 3:** Participants’ health status

Parameter	Description^a^	Clinical examples	IST (*n* = 31)	SET (*n* = 33)	CON (*n* = 36)
Health category					
A A1	Completely healthy; no medication		1 (3.23)	3 (9.09)	1 (2.78)
A2	Completely healthy; using only preventive medication	Hormonal replacement therapy, aspirin, ...	1 (3.23)	2 (6.06)	3 (8.33)
B B1	Functioning normally; presence of stabilized, non-cardiovascular disease; absence of cardiovascular abnormalities	Treated hypothyroidism, stable diabetes, ...	14 (45.16)	12 (36.36)	9 (25.00)
B2	Functioning normally; using medication with cardiovascular effect, no overt cardiovascular disease other than normalized arterial hypertension	Arterial hypertension, β blocking agent, ...	12 (38.71)	11 (33.33)	17 (47.22)
C	(history of) cardio-vascular pathology or abnormal ECG.	Bundle branch block, angina, CABG, ...	3 (9.67)	5 (15.16)	6 (16.67)
D	Presenting signs of acute or active disease at the moment of examination	Bronchospasm, swollen joints, influenza, ...	/	/	/
Age category (years)					
65–74			27 (87.10)	28 (84.85)	30 (83.33)
≥ 75			4 (12.90)	5 (15.15)	6 (16.67)

Note: The values denote number (percentage). ^a^Status after questioning, physical examination, ECG, and laboratory examination of blood, serum & urine according to the SENIEUR protocol [41]; *CABG* coronary artery bypass graft, *IST* Intensive strength training, *SET* Strength endurance training; *CON* control

### Training protocol

The purpose of this study was to evaluate the effects of 6 weeks intensive strength training (3 × 10 repetitions at 80% 1RM), strength endurance training (2 × 30 repetitions at 40% 1RM) and flexibility training (control) on the composition of blood T-cell subtypes in older women (see Additional file 1: Table S7 for detailed description of exercise interventions). Training took place at the exercise facilities of the Brussels Health Campus of the Vrije Universiteit Brussel on Technogym™ (Technogym, Gambettola, Italy) and Matrix® (Matrix, Wisconsin, USA) single station cable-type devices (for detailed specifications see manufacturers’ websites: https://www.technogym.com/be/fr/ and https://nl.matrixfitness.com/nl/). All training sessions were supervised by trained instructors to minimize the risk for injury and to make sure the participants used the proper technique and weights and performed the exercise throughout the entire range of motion. Each training session, regardless of the allocated training group, started with a warm-up of 10 exercises - that included mobility and activating exercises for both the upper and lower limbs (such as circular movements with the limbs, but no treadmill or cyclo-ergometer) - intended to prepare the joints and muscles for the up-coming exercises. Each warm-up exercise was performed without external resistance for a total of 15 repetitions per extremity or per exercise, duration of the complete warm-up was approximately 5 to 10 min. Six exercises (seated chest press, seated leg press, seated hip abduction, seated hip adduction, seated low row, and seated vertical traction) consisting of intensive sub-maximal muscle contractions with both concentric and eccentric components were performed in the intensive strength training and strength endurance training intervention groups. There was no predetermined sequence of the 6 exercises within each training session, however, participants were instructed to alter exercises for upper and lower limbs. Participants were asked to exercise 2–3 times weekly, with a minimum of 1-day interval for recuperation. The exercise protocols for the intensive strength training and strength endurance training intervention groups were designed to be approximately equal in volume (% 1RM multiplied by the number of sets and repetitions). For the intensive strength training intervention group, the large muscle groups were trained at 3 sets of 10 consecutive repetitions at 80% of 1RM. For the strength endurance training intervention group, the exercises were designed similarly as for the intensive strength training group, but with less intensive muscle contractions and a higher number of repetitions (2 sets of 30 consecutive repetitions at 40% of 1RM). The rest between sets was minimum 1 min for the intensive strength training and strength endurance training groups. All exercise programs entailed an initial accommodation period of 2 weeks in which the target exercise intensity was progressively reached. Every 2 weeks, the individuals’ 1RM was determined and exercise loads were adapted accordingly. A minimum of 24 h of rest (i.e. no training) was scheduled before and after a 1RM test. For the 1RM determination the participant starts with a warming up of 20 repetitions at 30% of the estimated or previously assessed 1RM. Next, the participant is asked to perform 1 repetition at 70% of the estimated or previously assessed 1RM. This step is repeated with increasing load until the participant is unable to perform the exercise correctly in full range of motion. The 1RM was considered as the highest load at which the participant was able to perform the exercise correctly in full range of motion. The load corresponding to 1RM was reached in maximum 4 to 5 steps.

The control group performed a “placebo” flexibility training consisting of 3 sets of sustained (30 s) passive, static stretching exercises of the large muscle groups (for adherence purpose). Stretching exercises act principally by applying mechanical tension on the muscles and tendons, leading to improved range of motion [44]. Therefore, this type of exercise - which mainly induces a passive load on the muscles and tendons without muscle contractions or cardiovascular challenge - was chosen as a control intervention.

### Anthropometric measurements

Weight was measured using a SECA balance, which was regularly calibrated to the nearest 0,1 kg. Height was determined using a SECA measuring rod to the nearest 0,1 cm. Body mass index was calculated - using the measurements of height and weight - as weight divided by height squared (weight (kg)/ height^2^ (m^2^)).

### Flow cytometry analysis

Venous blood specimens were collected before and after 6 weeks (24 h–48 h after the last training session) in the morning for serum (stored at − 80 °C until analysis) and for EDTA anticoagulated blood. Peripheral blood leucocytes were recovered as described previously [3]. Briefly, EDTA blood was exposed to lysis buffer for 10 min. After lysing the red blood cells, the blood leucocytes were centrifuged at 900×g for 4 min at room temperature. Thereafter, the cells were isolated, washed twice in PBS containing 1% BSA at 900×g for 3 min, and re-suspended in 200 μl PBS containing 1% BSA.

Antibodies were initially titrated to determine the optimal conditions for flow cytometry analysis before staining. About 5 × 10^5^ cells were stained with 3 μL each of PE-CY5-labelled anti-CD8 (Becton Dickinson, San Jose, CA, USA), PE-CY7-labelled anti-CD3 (Biolegend, San Diego, CA, USA), FITC-labelled anti-CD28 (Biolegend, San Diego, CA, USA), Dazzel-labelled anti-CD45 and PE-labelled anti-CD57 (Biolegend, San Diego, CA, USA). After 20 min of incubation at room temperature in the dark, cells were washed at 900×g for 3 min, and 500 μL of FACS flow solution (Becton Dickinson, San Jose, CA, USA) were added.

The labelled samples were analysed with a Coulter FC 500 flow cytometer (Beckman Coulter, Fullerton, CA, USA). Data acquisition was performed using the Coulter CXP software (Epics). The lymphocyte subpopulation was gated according to size and granularity in the forward vs. side scattergram, thereby excluding dead cells. Fluorescence-minus-one controls were used to distinguish positive from negative events and the various lymphocyte clusters were identified according to their expression of a combination of surface markers (see Additional file 2: Figures S2A-D for the gating procedure and representative dot plots for the delineation of T-cell sub-populations per intervention group). The expression (or non-expression) of CD28 and CD57 are particularly useful in distinguishing between subsets of differentiated T-cells. Based on these surface markers, CD8– and CD8+ T-cells were separated into four distinct sub-populations including CD28 + CD57–, CD28–CD57–, CD28–CD57+ and CD28 + CD57+. We used the terminologies naive (CD28 + CD57–, consisting predominantly of naive T-cells and perhaps some early differentiated T-cells), memory (CD28–CD57–), and senescence-prone (CD28–CD57+ and CD28 + CD57+) phenotypes to define the distinct subsets as previously described [3]. Absolute blood counts were measured using a dual platform methodology (flow cytometry and the Cell-Dyn Sapphire haematology analyser (Abbott Diagnostics Division, Santa Clara, CA)).

### Serum CMV IgG and CRP determination

Serum levels of CMV IgG were measured by a chemiluminescent microparticle immunoassay on the ARCHITECT iSystem (Abbott Diagnostics, Abbott Park, Ireland) with an assay sensitivity and specificity of 100 and 99%, respectively. Assays were regarded as positive if they had concentrations of 6.0 arbitrary units (AU)/mL or greater and negative if they had concentrations of less than 6.0 AU/mL. The detection limit of 6 AU/mL was based on the indications from the manufacturer of the CMV IgG kit. The intra-assay and inter-assay coefficients of variation ranged from 4.39 to 5.67% and from 4.87 to 6.17%, respectively. CRP was quantified by immunonephelometry using the high sensitivity CRP kit obtained from Dade Behring (Marburg GmbH, Germany). For CRP determination, the limit of detection was 0.175 mg/L and the intra-assay and inter-assay coefficients of variation ranged from 3.1 to 4.4% and from 2.5 to 5.7%, respectively. All reagents were applied according to the manufacturers’ instructions.

### Statistical analyses

Data distribution was tested by using the Kolmogorov-Smirnov Goodness of Fit test. Most of the parameters did not follow normal distribution even after log-transformation and therefore, nonparametric tests were applied during analysis. The Wilcoxon’s Signed Rank test, Kruskal-Wallis test and Mann-Whitney U test were used for continuous variables. Comparisons between categorical variables were performed using the chi-square test or Fisher exact test, where appropriate. Associations were explored by Spearman’s bivariate correlation test. The Kruskal-Wallis test was employed to test for difference among the intervention groups. When a significant difference was detected, between-group pairwise comparisons were performed using the Mann-Whitney U test. Wilcoxon’s Signed Rank test was applied for assessing time-effects. Changes after 6 weeks intervention were calculated and compared among the intensive strength training, strength endurance training and the control groups using Kruskal-Wallis test (and Mann-Whitney U test for post-hoc testing). Statistical analysis was performed using IBM SPSS version 24.0. Statistical significance was set a priori at two-sided *p* < 0.05.

## Additional files

Additional file 1: **Table S1.** Linear regression analysis of the association between the levels of baseline CMV IgG and the absolute counts of the senescence-prone T-cells, adjusted for age. Note: CMV = cytomegalovirus; SEB = standard error of the unstandardized regression coefficient. **Table S2.** Linear regression analysis of the association between the levels of baseline CMV IgG and the proportion of the senescence-prone T-cells, adjusted for age. Note: CMV = cytomegalovirus; SEB = standard error of the unstandardized regression coefficient. **Table S3.** Percentage and absolute counts of T-cell subsets at baseline in the different intervention groups with respect to CMV serostatus. Note: The values denote median (Interquartile range). CMV = cytomegalovirus; SPC = senescence-prone cells; IST = intensive strength training; SET = strength-endurance training; CON = control. T-cell subsets were expressed as percentages within the CD3 + CD8+ or CD3 + CD8− T-cells or absolute number of cells in peripheral blood (cells/μL). ^a^Results of Kruskal-Wallis test. **Table S4.** Training-induced changes in the absolute counts of CD8− T-cell phenotypes at 6 weeks compared to baseline among the different intervention groups in CMV seropositive participants. **Table S5.** Training-induced changes in the absolute counts of T-cell subsets among the different intervention groups in CMV seronegative participants. **Table S6.** Training-induced changes in the percentage of T-cell subsets among the different intervention groups in CMV seronegative participants. **Table S7.** Detailed description of exercise interventions. Note: 1RM = one repetition maximum. (ZIP 102 kb)

Additional file 2: **Figure S1.** Training-induced changes in the absolute counts of naive and memory phenotypes stratified for CMV. Note: Data are median values with error bars representing 95%CI. CMV = cytomegalovirus. *p < 0.05, increased significantly after exercise compared to baseline. **Figure S2.** The gating procedure and representative dot plots for the delineation of T-cell sub-populations by flow cytometry. Figure S2A (gating strategy), Figure S2B (representative plots for the strength endurance training group), Figure S2C (representative plots for the intensive strength training group) and Figure S2D (representative plots for the control group). (ZIP 625 kb)

## Data Availability

All data generated or analysed during this study are included in this published article (and its supplementary information files).

## Abbreviations

1RM: 1 repetition maximum
BMI: Body mass index
CABG: Coronary artery bypass graft
CMV: Cytomegalovirus
CRP: C-reactive protein
IL-6: Interleukin-6
PCR: Polymerase chain reaction
SPC: Senescence-prone cells
TNF-α: Tumour necrosis factor-α
