# Supplementary material for: Six weeks of strength endurance training decreases circulating senescence-prone T-lymphocytes in cytomegalovirus seropositive but not seronegative older women
Source: Immun Ageing. 2019 Jul 25;16:17. doi: 10.1186/s12979-019-0157-8 (PMC6657061; doi:10.1186/s12979-019-0157-8)
Supplement: Supplementary file 1 — Table S1. Linear regression analysis of the association between the levels of baseline CMV IgG and the absolute counts of the senescence-prone T-cells, adjusted for age. Note: CMV = cytomegalovirus; SEB = standard error of the unstandardized regression coefficient. Table S2. Linear regression analysis of the association between the levels of baseline CMV IgG and the proportion of the senescence-prone T-cells, adjusted for age. Note: CMV = cytomegalovirus; SEB = standard error of the unstandardized regression coefficient. Table S3. Percentage and absolute counts of T-cell subsets at baseline in the different intervention groups with respect to CMV serostatus. Note: The values denote median (Interquartile range). CMV = cytomegalovirus; SPC = senescence-prone cells; IST = intensive strength training; SET = strength-endurance training; CON = control. T-cell subsets were expressed as percentages within the CD3 + CD8+ or CD3 + CD8− T-cells or absolute number of cells in peripheral blood (cells/μL). aResults of Kruskal-Wallis test. Table S4. Training-induced changes in the absolute counts of CD8− T-cell phenotypes at 6 weeks compared to baseline among the different intervention groups in CMV seropositive participants. Table S5. Training-induced changes in the absolute counts of T-cell subsets among the different intervention groups in CMV seronegative participants. Table S6. Training-induced changes in the percentage of T-cell subsets among the different intervention groups in CMV seronegative participants. Table S7. Detailed description of exercise interventions. Note: 1RM = one repetition maximum. (ZIP 102 kb) [file 12979_2019_157_MOESM1_ESM.zip › Supplementary Table S1 R3.docx]

| **CD8+ T-cells** | | | | | **CD8−T-cells** | | | | |
| --- | --- | --- | --- | --- | --- | --- | --- | --- | --- |
|  | **Unstandardized**  **Coefficients** | | **standardized**  **Coefficients** |  |  | **Unstandardized**  **Coefficients** | | **standardized**  **Coefficients** |  |
| **Model** | **B** | **SEB** | **β** | **p** | **Model** | **B** | **SEB** | **β** | **p** |
| **CD8+CD57+** |  |  |  |  | **CD8−CD57+** |  |  |  |  |
| **Constant** | -24.547 | 76.963 |  | 0.750 | **Constant** | -32.320 | 26.594 |  | 0.227 |
| **Age** | 0.582 | 1.110 | 0.054 | 0.602 | **Age** | 0.498 | 0.384 | 0.127 | 0.198 |
| **CMV IgG** | 0.117 | 0.041 | 0.296 | **0.005** | **CMV IgG** | 0.053 | 0.014 | 0.363 | **<0.001** |
|  |  |  |  |  |  |  |  |  |  |
| **CD8+CD28−CD57+** |  |  |  |  | **CD8−CD28−CD57+** |  |  |  |  |
| **Constant** | -34.507 | 73.691 |  | 0.641 | **Constant** | -40.147 | 24.803 |  | 0.109 |
| **Age** | 0.709 | 1.063 | 0.069 | 0.506 | **Age** | 0.606 | 0.358 | 0.167 | 0.094 |
| **CMV IgG** | 0.112 | 0.039 | 0.295 | **0.005** | **CMV IgG** | 0.043 | 0.013 | 0.317 | **0.002** |
|  |  |  |  |  |  |  |  |  |  |
| **CD8+CD28+CD57+** |  |  |  |  | **CD8−CD28+CD57+** |  |  |  |  |
| **Constant** | 9.960 | 6.255 |  | 0.115 | **Constant** | 7.827 | 6.416 |  | 0.226 |
| **Age** | -0.128 | 0.090 | -0.150 | 0.161 | **Age** | -0.108 | 0.093 | -0.121 | 0.246 |
| **CMV IgG** | 0.005 | 0.003 | 0.172 | 0.108 | **CMV IgG** | 0.010 | 0.003 | 0.306 | **0.004** |

**Table S1** Linear regression analysis of the association between the levels of baseline CMV IgG and the absolute counts of the senescence-prone T-cells, adjusted for age
