# Supplementary material for: Six weeks of strength endurance training decreases circulating senescence-prone T-lymphocytes in cytomegalovirus seropositive but not seronegative older women
Source: Immun Ageing. 2019 Jul 25;16:17. doi: 10.1186/s12979-019-0157-8 (PMC6657061; doi:10.1186/s12979-019-0157-8)
Supplement: Supplementary file 1 — Table S1. Linear regression analysis of the association between the levels of baseline CMV IgG and the absolute counts of the senescence-prone T-cells, adjusted for age. Note: CMV = cytomegalovirus; SEB = standard error of the unstandardized regression coefficient. Table S2. Linear regression analysis of the association between the levels of baseline CMV IgG and the proportion of the senescence-prone T-cells, adjusted for age. Note: CMV = cytomegalovirus; SEB = standard error of the unstandardized regression coefficient. Table S3. Percentage and absolute counts of T-cell subsets at baseline in the different intervention groups with respect to CMV serostatus. Note: The values denote median (Interquartile range). CMV = cytomegalovirus; SPC = senescence-prone cells; IST = intensive strength training; SET = strength-endurance training; CON = control. T-cell subsets were expressed as percentages within the CD3 + CD8+ or CD3 + CD8− T-cells or absolute number of cells in peripheral blood (cells/μL). aResults of Kruskal-Wallis test. Table S4. Training-induced changes in the absolute counts of CD8− T-cell phenotypes at 6 weeks compared to baseline among the different intervention groups in CMV seropositive participants. Table S5. Training-induced changes in the absolute counts of T-cell subsets among the different intervention groups in CMV seronegative participants. Table S6. Training-induced changes in the percentage of T-cell subsets among the different intervention groups in CMV seronegative participants. Table S7. Detailed description of exercise interventions. Note: 1RM = one repetition maximum. (ZIP 102 kb) [file 12979_2019_157_MOESM1_ESM.zip › Supplementary Table S2 R3.docx]

| **CD8+ T-cells** | | | | | **CD8−T-cells** | | | | |
| --- | --- | --- | --- | --- | --- | --- | --- | --- | --- |
|  | **Unstandardized**  **Coefficients** | | **standardized**  **Coefficients** |  |  | **Unstandardized**  **Coefficients** | | **standardized**  **Coefficients** |  |
| **Model** | **B** | **SEB** | **β** | **p** | **Model** | **B** | **SEB** | **β** | **p** |
| **CD8+CD57+** |  |  |  |  | **CD8−CD57+** |  |  |  |  |
| **Constant** | -15.889 | 19.853 |  | 0.426 | **Constant** | -4.975 | 4.300 |  | 0.250 |
| **Age** | 0.323 | 0.286 | 0.118 | 0.262 | **Age** | 0.075 | 0.062 | 0.117 | 0.233 |
| **CMV IgG** | 0.021 | 0.010 | 0.209 | **0.048** | **CMV IgG** | 0.009 | 0.002 | 0.383 | **<0.001** |
|  |  |  |  |  |  |  |  |  |  |
| **CD8+CD28−CD57+** |  |  |  |  | **CD8−CD28−CD57+** |  |  |  |  |
| **Constant** | -18.061 | 19.086 |  | 0.346 | **Constant** | -6.060 | 3.930 |  | 0.126 |
| **Age** | 0.347 | 0.275 | 0.131 | 0.210 | **Age** | 0.090 | 0.057 | 0.154 | 0.118 |
| **CMV IgG** | .021 | 0.010 | 0.212 | **0.044** | **CMV IgG** | 0.008 | 0.002 | 0.350 | **0.001** |
|  |  |  |  |  |  |  |  |  |  |
| **CD8+CD28+CD57+** |  |  |  |  | **CD8−CD28+CD57+** |  |  |  |  |
| **Constant** | 2.171 | 1.691 |  | 0.202 | **Constant** | 1.085 | 0.983 |  | 0.272 |
| **Age** | -0.024 | 0.024 | -0.106 | 0.328 | **Age** | -0.015 | 0.014 | -0.109 | 0.295 |
| **CMV IgG** | 0.000 | 0.001 | 0.049 | 0.649 | **CMV IgG** | 0.002 | 0.001 | 0.303 | **0.004** |

**Table S2** Linear regression analysis of the association between the levels of baseline CMV IgG and the proportion of the senescence-prone T-cells, adjusted for age
