# Supplementary material for: Six weeks of strength endurance training decreases circulating senescence-prone T-lymphocytes in cytomegalovirus seropositive but not seronegative older women
Source: Immun Ageing. 2019 Jul 25;16:17. doi: 10.1186/s12979-019-0157-8 (PMC6657061; doi:10.1186/s12979-019-0157-8)
Supplement: Supplementary file 1 — Table S1. Linear regression analysis of the association between the levels of baseline CMV IgG and the absolute counts of the senescence-prone T-cells, adjusted for age. Note: CMV = cytomegalovirus; SEB = standard error of the unstandardized regression coefficient. Table S2. Linear regression analysis of the association between the levels of baseline CMV IgG and the proportion of the senescence-prone T-cells, adjusted for age. Note: CMV = cytomegalovirus; SEB = standard error of the unstandardized regression coefficient. Table S3. Percentage and absolute counts of T-cell subsets at baseline in the different intervention groups with respect to CMV serostatus. Note: The values denote median (Interquartile range). CMV = cytomegalovirus; SPC = senescence-prone cells; IST = intensive strength training; SET = strength-endurance training; CON = control. T-cell subsets were expressed as percentages within the CD3 + CD8+ or CD3 + CD8− T-cells or absolute number of cells in peripheral blood (cells/μL). aResults of Kruskal-Wallis test. Table S4. Training-induced changes in the absolute counts of CD8− T-cell phenotypes at 6 weeks compared to baseline among the different intervention groups in CMV seropositive participants. Table S5. Training-induced changes in the absolute counts of T-cell subsets among the different intervention groups in CMV seronegative participants. Table S6. Training-induced changes in the percentage of T-cell subsets among the different intervention groups in CMV seronegative participants. Table S7. Detailed description of exercise interventions. Note: 1RM = one repetition maximum. (ZIP 102 kb) [file 12979_2019_157_MOESM1_ESM.zip › Supplementary Table S3 R3.docx]

|  | | **CMV+ (n=63)** | | | | **CMV− (n= 34)** | | | |
| --- | --- | --- | --- | --- | --- | --- | --- | --- | --- |
| **T-cell subset** | | **IST (n=24)** | **SET (n=19)** | **CON (n=20)** | **p** ^a^ | **IST (n=06)** | **SET (n=13)** | **CON (n=15)** | **p** ^a^ |
| **CD8+ T-cells** | |  |  |  |  |  |  |  |  |
| CD8+CD28+CD57− (naïve) | cells/µL | 188.17 (140.92) | 154.60 (120.68) | 148.43 (70.48) | 0.843 | 145.60 (123.85) | 129.02 (100.36) | 136.05 (71.52) | 0.902 |
|  | % | 51.10 (25) | 61.10 (20.20) | 50.85 (31.53) | 0.388 | 72.50 (34.47) | 76.60 (22.40) | 68.00 (30.10) | 0.887 |
| CD8+CD28−CD57− (memory) | cells/µL | 110.00 (152.50) | 70.00 (100.00) | 90.00 (67.50) | 0.579 | 60.00 (112.50) | 30.00 (40.00) | 40.00 (40.00) | 0.986 |
|  | % | 28.00 (23.58) | 29.50 (19.20) | 32.00 (23.17) | 0.793 | 22.70 (34.38) | 19.30 (15.45) | 20.70 (18.70) | 0.985 |
| CD8+CD57+ (SPC) | cells/µL | 27.97 (45.80) | 27.71 (34.77) | 23.22 (38.23) | 0.654 | 19.05 (16.19) | 6.40 (14.53) | 3.46 (24.96) | 0.343 |
|  | % | 6.00 (15.50) | 6.90 (12.20) | 7.45 (12.38) | 0.798 | 5.40 (4.70) | 4.00 (8.70) | 1.85 (9.95) | 0.359 |
| CD8+CD28−CD57+ (SPC) | cells/µL | 26.99 (45.50) | 27.71 (33.83) | 22.79 (37.58) | 0.644 | 17.28 (14.36) | 5.15 (13.54) | 2.48 (21.08) | 0.282 |
|  | % | 5.80 (15.50) | 6.80 (11.20) | 6.65 (11.73) | 0.808 | 4.95 (4.13) | 3.70 (8.15) | 1.20 (8.70) | 0.364 |
| CD8+CD28+CD57+ (SPC) | cells/µL | 0.83 (1.36) | 0.46 (1.09) | 0.58 (1.49) | 0.566 | 1.05 (2.26) | 0.84 (1.15) | 0.76 (1.80) | 0.763 |
|  | % | 0.30 (0.40) | 0.10 (0.40) | 0.20 (0.48) | 0.672 | 0.45 (0.93) | 0.50 (0.60) | 0.30 (0.92) | 0.772 |
| **CD8− T-cells** | |  |  |  |  |  |  |  |  |
| CD8−CD28+CD57− (naïve) | cells/µL | 616.90 (426.18) | 664.54 (280.26) | 650.41 (402.95) | 0.718 | 580.44 (294.33) | 656.32 (450.04) | 641.26 (317.52) | 0.463 |
|  | % | 96.70 (4.10) | 96.50 (6.50) | 95.60 (5.45) | 0.883 | 99.45 (0.60) | 99.40 (0.90) | 99.20 (1.30) | 0.977 |
| CD8−CD28−CD57− (memory) | cells/µL | 10.00 (20.00) | 20.00 (20.00) | 20.00 (30.00) | 0.708 | 0.00 (0.00) | 0.00 (5.00) | 0.00 (10.00) | 0.215 |
|  | % | 1.65 (4.45) | 2.00 (4.70) | 2.75 (3.70) | 0.888 | 0.30 (0.25) | 0.50 (0.80) | 0.60 (1.10) | 0.609 |
| CD8−CD57+ (SPC) | cells/µL | 6.71 (18.08) | 6.55 (12.35) | 8.70 (16.21) | 0.998 | 0.85 (2.02) | 1.10 (1.95) | 0.65 (1.74) | 0.698 |
|  | % | 0.95 (2.35) | 0.80 (1.90) | 1.00 (2.63) | 0.899 | 0.10 (0.30) | 0.10 (0.30) | 0.10 (0.50) | 0.803 |
| CD8−CD28−CD57+ (SPC) | cells/µL | 5.02 (11.95) | 6.12 (9.28) | 7.67 (13.12) | 0.968 | 0.26 (1.72) | 0.00 (1.23) | 0.00 (0.82) | 0.961 |
|  | % | 0.85 (1.45) | 0.70 (1.80) | 0.90 (2.10) | 0.986 | 0.05 (0.28) | 0.00 (0.20) | 0.00 (0.20) | 0.946 |
| CD8−CD28+CD57+ (SPC) | cells/µL | 0.62 (2.40) | 0.87 (2.50) | 0.92 (1.67) | 0.726 | 0.58 (0.94) | 0.57 (0.96) | 0.46 (0.91) | 0.836 |
|  | % | 0.10 (0.38) | 0.10 (0.30) | 0.10 (0.35) | 0.546 | 0.10 (0.05) | 0.10 (0.10) | 0.10 (0.10) | 0.735 |

**Table S3** Absolute counts and percentage of T-cell subsets at baseline in the different intervention groups with respect to CMV serostatus.

Note: CMV = cytomegalovirus; SPC = senescence-prone cells; IST = intensive strength training; SET = strength-endurance training; CON = control. T-cell subsets were expressed as percentages within the CD3+CD8+ or CD3+CD8− T-cells or absolute number of cells in peripheral blood (cells/µL). ^a^ Results of Kruskal-Wallis test.
