# Supplementary material for: Six weeks of strength endurance training decreases circulating senescence-prone T-lymphocytes in cytomegalovirus seropositive but not seronegative older women
Source: Immun Ageing. 2019 Jul 25;16:17. doi: 10.1186/s12979-019-0157-8 (PMC6657061; doi:10.1186/s12979-019-0157-8)
Supplement: Supplementary file 1 — Table S1. Linear regression analysis of the association between the levels of baseline CMV IgG and the absolute counts of the senescence-prone T-cells, adjusted for age. Note: CMV = cytomegalovirus; SEB = standard error of the unstandardized regression coefficient. Table S2. Linear regression analysis of the association between the levels of baseline CMV IgG and the proportion of the senescence-prone T-cells, adjusted for age. Note: CMV = cytomegalovirus; SEB = standard error of the unstandardized regression coefficient. Table S3. Percentage and absolute counts of T-cell subsets at baseline in the different intervention groups with respect to CMV serostatus. Note: The values denote median (Interquartile range). CMV = cytomegalovirus; SPC = senescence-prone cells; IST = intensive strength training; SET = strength-endurance training; CON = control. T-cell subsets were expressed as percentages within the CD3 + CD8+ or CD3 + CD8− T-cells or absolute number of cells in peripheral blood (cells/μL). aResults of Kruskal-Wallis test. Table S4. Training-induced changes in the absolute counts of CD8− T-cell phenotypes at 6 weeks compared to baseline among the different intervention groups in CMV seropositive participants. Table S5. Training-induced changes in the absolute counts of T-cell subsets among the different intervention groups in CMV seronegative participants. Table S6. Training-induced changes in the percentage of T-cell subsets among the different intervention groups in CMV seronegative participants. Table S7. Detailed description of exercise interventions. Note: 1RM = one repetition maximum. (ZIP 102 kb) [file 12979_2019_157_MOESM1_ESM.zip › Supplementary Table S4 R3.docx]

**Table S4** Training-induced changes in the absolute counts of naïve CD8− T-cells at 6 weeks compared to baseline among the different

intervention groups in CMV seropositive participants.

| **T-cell subset** | **IST (n=21)** | **SET (n=15)** | **CON (n=19)** | **Time effect** ^a^ | **Time * group effect** ^b^ |
| --- | --- | --- | --- | --- | --- |
|  |  |  |  |  |  |
| **CD8− T-cells** |  |  |  |  |  |
| CD8−CD28+CD57− (naive) |  |  |  |  |  |
| Baseline | 597.25 (380.07) | 575.11 (280.26) | 650.27 (416.79) | **0.047** | 0.647 |
| 6 weeks | 725.99 (484.04) | 596.49 (352.35) | 711.33 (388.17) |  |  |
| CD8−CD28−CD57− (memory) |  |  |  |  |  |
| Baseline | 10.00 (30.00) | 20.00 (20.00) | 20.00 (30.00) | 0.406 | 0.945 |
| 6 weeks | 20.00 (20.00) | 10.00 (20.00) | 20.00 (20.00) |  |  |
| CD8−CD57+ (SPC) |  |  |  |  |  |
| Baseline | 6.92 (18.65) | 6.55 (12.37) | 9.00 (17.03) | **0.037** | 0.560 |
| 6 weeks | 6.70 (9.16) | 3.18 (6.83) * | 7.08 (15.03) |  |  |
|  |  |  |  |  |  |

Note: The values denote median (Interquartile range) of the absolute number of cells per µL blood; CMV, cytomegalovirus; IST= intensive strength training; SET= strength-endurance training; CON= control; ^a^ Wilcoxon signed-rank test for changes between baseline and 6 weeks; ^b^ Kruskal-Wallis test for changes between baseline and 6 weeks - a real numerical value was computed for each individual - among the 3 groups of training, *p<0.05 significantly different from baseline within group.
