# Supplementary material for: Six weeks of strength endurance training decreases circulating senescence-prone T-lymphocytes in cytomegalovirus seropositive but not seronegative older women
Source: Immun Ageing. 2019 Jul 25;16:17. doi: 10.1186/s12979-019-0157-8 (PMC6657061; doi:10.1186/s12979-019-0157-8)
Supplement: Supplementary file 1 — Table S1. Linear regression analysis of the association between the levels of baseline CMV IgG and the absolute counts of the senescence-prone T-cells, adjusted for age. Note: CMV = cytomegalovirus; SEB = standard error of the unstandardized regression coefficient. Table S2. Linear regression analysis of the association between the levels of baseline CMV IgG and the proportion of the senescence-prone T-cells, adjusted for age. Note: CMV = cytomegalovirus; SEB = standard error of the unstandardized regression coefficient. Table S3. Percentage and absolute counts of T-cell subsets at baseline in the different intervention groups with respect to CMV serostatus. Note: The values denote median (Interquartile range). CMV = cytomegalovirus; SPC = senescence-prone cells; IST = intensive strength training; SET = strength-endurance training; CON = control. T-cell subsets were expressed as percentages within the CD3 + CD8+ or CD3 + CD8− T-cells or absolute number of cells in peripheral blood (cells/μL). aResults of Kruskal-Wallis test. Table S4. Training-induced changes in the absolute counts of CD8− T-cell phenotypes at 6 weeks compared to baseline among the different intervention groups in CMV seropositive participants. Table S5. Training-induced changes in the absolute counts of T-cell subsets among the different intervention groups in CMV seronegative participants. Table S6. Training-induced changes in the percentage of T-cell subsets among the different intervention groups in CMV seronegative participants. Table S7. Detailed description of exercise interventions. Note: 1RM = one repetition maximum. (ZIP 102 kb) [file 12979_2019_157_MOESM1_ESM.zip › Supplementary Table S5 R3.docx]

| **Table S5** Training-induced changes in the absolute counts of T-cell subsets among the different intervention groups in CMV seronegative participants | | | | | | | |
| --- | --- | --- | --- | --- | --- | --- | --- |
| **T-cell subset** | | **IST (n=05)** | **SET (n=11)** | **CON (n=13)** | | **Time effect** ^a^ | **Time * group effect** ^b^ |
| **CD8+ T-cells** | | | | | | | |
| CD8+CD28+CD57− (naive) | | | | | | | |
| Baseline | | 174.67 (135.65) | 101.50 (104.41) | | 137.79 (81.84) | 0.443 | 0.763 |
| 6 weeks | | 155.37 (127.81) | 100.88 (47.00) | | 118.32 (113.84) |  |  |
| CD8+CD28−CD57− (memory) | | | | | | | |
| Baseline | | 20.00 (115.00) | 30.00 (60.00) | 40.00 (35.00) | | 0.363 | 0.645 |
| 6 weeks | | 20.00 (65.00) | 30.00 (40.00) | 40.00 (25.00) | |  |  |
| CD8+CD57+ (SPC) | | | | | | | |
| Baseline | | 18.37 (16.15) | 5.85 (20.56) | 2.65 (5.16) | | 0.428 | 0.276 |
| 6 weeks | | 5.20 (29.23) | 7.54 (16.95) | 2.39 (6.62) | |  |  |
| CD8+CD28−CD57+ (SPC) | | |  |  | |  |  |
| Baseline | 17.16 (14.18) | | 5.21 (18.77) | 1.80 (4.22) | | 0.532 | 0.234 |
| 6 weeks | 4.68 (24.87) | | 6.79 (14.85) | 1.89 (5.81) | |  |  |
| CD8+CD28+CD57+ (SPC) | | |  |  | |  |  |
| Baseline | | 1.14 (2.17) | 0.50 (1.05) | 0.55 (1.49) | | 0.770 | 0.712 |
| 6 weeks | | 0.98 (4.59) | 0.51 (1.22) | 0.56 (1.20) | |  |  |
| **CD8− T-cells** | | | | | | | |
| CD8−CD28+CD57− (naive) | | | | | | | |
| Baseline | | 630.58 (464.60) | 655.34 (584.74) | 642.57 (280.73) | | 0.144 | 0.352 |
| 6 weeks | | 740.43 (255.17) | 616.20 (271.45) | 593.40 (411.41) | |  |  |
| CD8−CD28−CD57− (memory) | | | | | | | |
| Baseline | | 0.00 (0.00) | 0.00 (10.00) | 0.00 (10.00) | | 0.705 | 0.891 |
| 6 weeks | | 0.00 (0.00) | 0.00 (10.00) | 0.00 (10.00) | |  |  |
| CD8−CD57+ (SPC) | | | | | | | |
| Baseline | | 1.06 (3.38) | 1.10 (2.65) | 0.65 (1.62) | | 0.758 | 0.465 |
| 6 weeks | | 1.02 (3.79) | 1.22 (3.69) | 0.00 (2.30) | |  |  |
| CD8−CD28−CD57+ (SPC) | | |  |  | |  |  |
| Baseline | | 0.00 (2.90) | 0.00 (1.59) | 0.00 (1.25) | | 0.507 | 0.306 |
| 6 weeks | | 0.51 (3.00) | 0.00 (2.46) | 0.00 (1.82) | |  |  |
| CD8−CD28+CD57+ (SPC) | | |  |  | |  |  |
| Baseline | | 0.63 (0.80) | 0.55 (1.06) | 0.28 (0.83) | | 0.903 | 0.776 |
| 6 weeks | | 0.65 (0.86) | 0.61 (1.22) | 0.00 (0.61) | |  |  |
| Note: The values denote median (Interquartile range). CMV = cytomegalovirus; SPC = senescence-prone cells; IST = intensive strength training; SET = strength-endurance training; CON = control. Subset values were expressed as absolute number of cells in peripheral blood (cells/µL). ^a^ Wilcoxon signed-rank test for changes between baseline and 6 weeks for the whole CMV negative cohort; ^b^ Kruskal-Wallis test for changes between baseline and 6 weeks - a real numerical value was computed for each individual - among the 3 groups of training. | | | | | | | |
