# Supplementary material for: Six weeks of strength endurance training decreases circulating senescence-prone T-lymphocytes in cytomegalovirus seropositive but not seronegative older women
Source: Immun Ageing. 2019 Jul 25;16:17. doi: 10.1186/s12979-019-0157-8 (PMC6657061; doi:10.1186/s12979-019-0157-8)
Supplement: Supplementary file 1 — Table S1. Linear regression analysis of the association between the levels of baseline CMV IgG and the absolute counts of the senescence-prone T-cells, adjusted for age. Note: CMV = cytomegalovirus; SEB = standard error of the unstandardized regression coefficient. Table S2. Linear regression analysis of the association between the levels of baseline CMV IgG and the proportion of the senescence-prone T-cells, adjusted for age. Note: CMV = cytomegalovirus; SEB = standard error of the unstandardized regression coefficient. Table S3. Percentage and absolute counts of T-cell subsets at baseline in the different intervention groups with respect to CMV serostatus. Note: The values denote median (Interquartile range). CMV = cytomegalovirus; SPC = senescence-prone cells; IST = intensive strength training; SET = strength-endurance training; CON = control. T-cell subsets were expressed as percentages within the CD3 + CD8+ or CD3 + CD8− T-cells or absolute number of cells in peripheral blood (cells/μL). aResults of Kruskal-Wallis test. Table S4. Training-induced changes in the absolute counts of CD8− T-cell phenotypes at 6 weeks compared to baseline among the different intervention groups in CMV seropositive participants. Table S5. Training-induced changes in the absolute counts of T-cell subsets among the different intervention groups in CMV seronegative participants. Table S6. Training-induced changes in the percentage of T-cell subsets among the different intervention groups in CMV seronegative participants. Table S7. Detailed description of exercise interventions. Note: 1RM = one repetition maximum. (ZIP 102 kb) [file 12979_2019_157_MOESM1_ESM.zip › Supplementary Table S6 R3.docx]

| **Table S6** Training-induced changes in the percentage of T-cell subsets among the different intervention groups in CMV seronegative participants | | | | | | | | |
| --- | --- | --- | --- | --- | --- | --- | --- | --- |
| **T-cell subset** | | **IST (n=05)** | **SET (n=13)** | | | **CON (n=15)** | **Time effect** ^a^ | **Time * group effect** ^b^ |
| **CD8+ T-cells** | | | | | | | | |
| CD8+CD28+CD57− (naive) | | | | | | | | |
| Baseline | | 80.10 (28.75) | 76.60 (22.40) | | | 68.00 (30.10) | 0.695 | 0.337 |
| 6 weeks | | 78.60 (25.15) | 71.40 (20.15) | | | 67.30 (26.40) |  |  |
| CD8+CD28− CD57− (memory) | | | | | | | | |
| Baseline | | 15.50 (29.05) | 19.30 (15.45) | | | 20.70 (18.70) | 0.421 | 0.756 |
| 6 weeks | | 17.90 (20.80) | 21.00 (20.55) | | | 21.50 (22.80) |  |  |
| CD8+CD57+ (SPC) | | | | | | | | |
| Baseline | | 5.10 (3.55) | 3.75 (6.02) | | | 1.85 (9.95) | 0.133 | 0.538 |
| 6 weeks | | 2.90 (8.35) | 3.00 (7.28) | | | 2.30 (7.00) |  |  |
| CD8+CD28−CD57+ (SPC) | | |  | | |  |  |  |
| Baseline | 4.50 (3.40) | | | 3.20 (5.42) | 1.20 (8.70) | | 0.181 | 0.427 |
| 6 weeks | 2.50 (7.20) | | | 2.70 (6.75) | 1.75 (6.90) | |  |  |
| CD8+CD28+CD57+ (SPC) | | |  | | |  |  |  |
| Baseline | | 0.60 (0.95) | 0.40 (0.60) | | | 0.30 (0.92) | 0.441 | 0.897 |
| 6 weeks | | 0.80 (1.35) | 0.35 (0.58) | | | 0.35 (0.67) |  |  |
| **CD8− T-cells** | | | | | | | | |
| CD8−CD28+CD57− (naive) | | | | | | | | |
| Baseline | | 99.50 (0.55) | 99.40 (0.90) | | | 99.20 (1.30) | 0.068 | 0.713 |
| 6 weeks | | 99.40 (0.65) | 99.30 (1.10) | | | 99.30 (1.60) |  |  |
| CD8−CD28− CD57− (memory) | | | | | | | | |
| Baseline | | 0.30 (0.15) | 0.50 (0.80) | | | 0.60 (1.10) | 0.061 | 0.945 |
| 6 weeks | | 0.40 (0.30) | 0.50 (1.00) | | | 0.70 (1.00) |  |  |
| CD8−CD57+ (SPC) | | | | | | | | |
| Baseline | | 0.10 (0.50) | 0.10 (0.30) | | | 0.10 (0.50) | 0.611 | 0.538 |
| 6 weeks | | 0.20 (0.50) | 0.10 (0.50) | | | 0.00 (0.50) |  |  |
| CD8−CD28−CD57+ (SPC) | | |  | | |  |  |  |
| Baseline | | 0.00 (0.45) | 0.00 (0.20) | | | 0.00 (0.20) | 0.330 | 0.391 |
| 6 weeks | | 0.10 (0.40) | 0.00 (0.25) | | | 0.00 (0.20) |  |  |
| CD8−CD28+CD57+ (SPC) | | |  | | |  |  |  |
| Baseline | | 0.10 (0.05) | 0.10 (0.10) | | | 0.10 (0.10) | 0.765 | 0.802 |
| 6 weeks | | 0.10 (0.10) | 0.10 (0.20) | | | 0.00 (0.10) |  |  |
| Note: The values denote median (Interquartile range). CMV = cytomegalovirus; SPC = senescence-prone cells; IST = intensive strength training; SET = strength-endurance training; CON = control. T-cell subsets were expressed as percentages within the CD3+CD8+ or CD3+CD8− T-cells. ^a^ Wilcoxon signed-rank test for changes between baseline and 6 weeks for the whole CMV negative cohort; ^b^ Kruskal-Wallis test for changes between baseline and 6 weeks (a real numerical value was computed for each individual) among the 3 groups of training. | | | | | | | | |
