# Supplementary figures and images for: Six weeks of strength endurance training decreases circulating senescence-prone T-lymphocytes in cytomegalovirus seropositive but not seronegative older women
Source: Immun Ageing. 2019 Jul 25;16:17. doi: 10.1186/s12979-019-0157-8 (PMC6657061; doi:10.1186/s12979-019-0157-8)

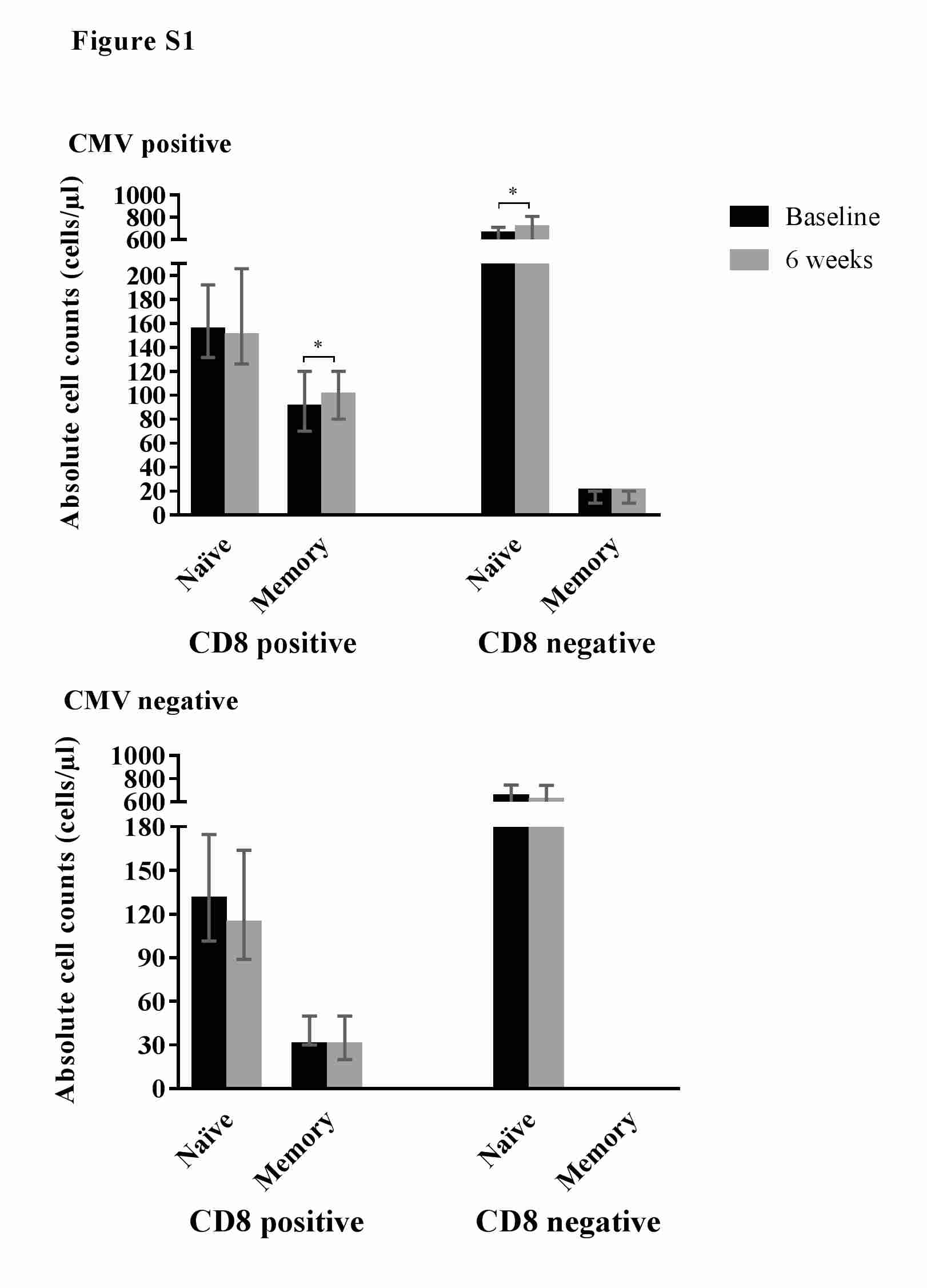

Supplement: Supplementary file 2 — Figure S1. Training-induced changes in the absolute counts of naive and memory phenotypes stratified for CMV. Note: Data are median values with error bars representing 95%CI. CMV = cytomegalovirus. *p < 0.05, increased significantly after exercise compared to baseline. Figure S2. The gating procedure and representative dot plots for the delineation of T-cell sub-populations by flow cytometry. Figure S2A (gating strategy), Figure S2B (representative plots for the strength endurance training group), Figure S2C (representative plots for the intensive strength training group) and Figure S2D (representative plots for the control group). (ZIP 625 kb) [file 12979_2019_157_MOESM2_ESM.zip › Supplementary Figure S1 R3.jpg]

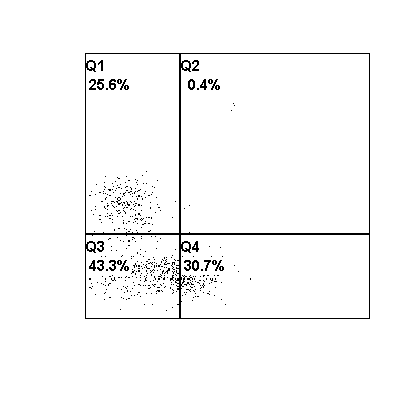

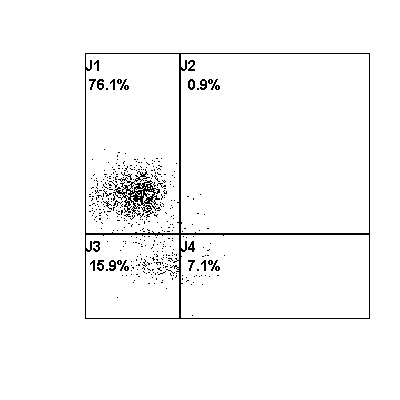

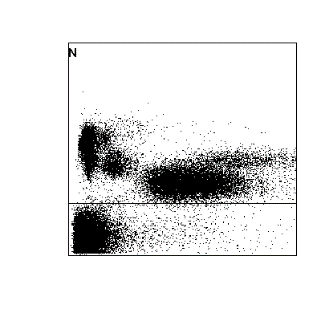

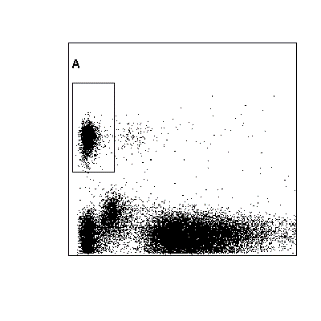

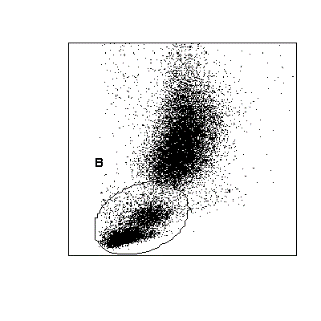

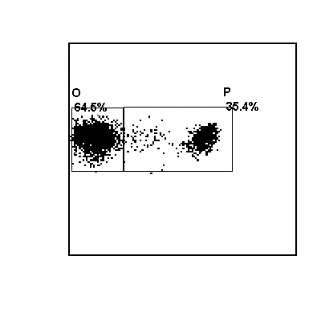


**CD45**

**CD3**

**CD3**

**SS**

**CD8**

**SS**

**SS**

**FS**

**CD57**

**CD57**

**CD28**

**CD28**

**CD8─**

**CD8+**

**Figure S2A**

Supplement: Supplementary file 2 — Figure S1. Training-induced changes in the absolute counts of naive and memory phenotypes stratified for CMV. Note: Data are median values with error bars representing 95%CI. CMV = cytomegalovirus. *p < 0.05, increased significantly after exercise compared to baseline. Figure S2. The gating procedure and representative dot plots for the delineation of T-cell sub-populations by flow cytometry. Figure S2A (gating strategy), Figure S2B (representative plots for the strength endurance training group), Figure S2C (representative plots for the intensive strength training group) and Figure S2D (representative plots for the control group). (ZIP 625 kb) [file 12979_2019_157_MOESM2_ESM.zip › Supplementary Figure S2A R3.docx]

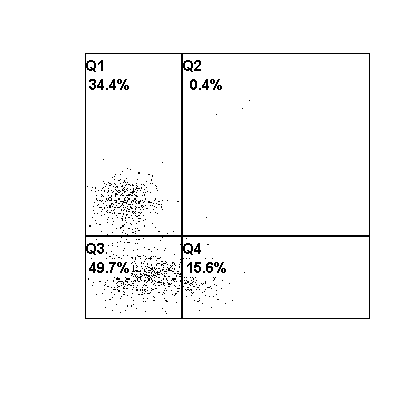

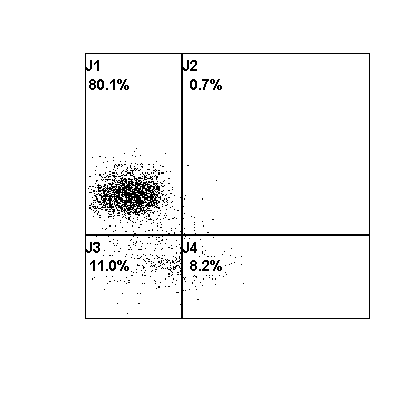

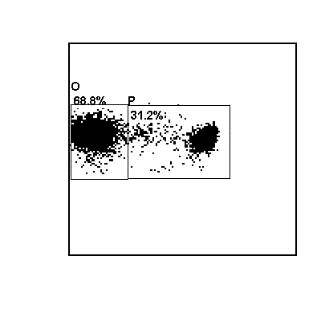

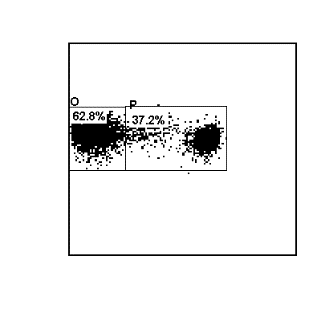

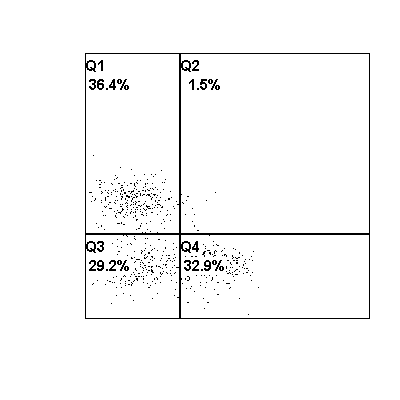

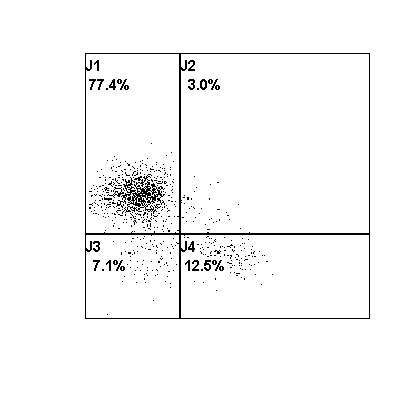


**CD3**

**CD8**

**CD57**

**CD57**

**CD28**

**CD28**

**CD8─**

**CD8+**

**CD3**

**CD8**

**CD57**

**CD57**

**CD28**

**CD28**

**CD8─**

**CD8+**

**Figure S2B**

**Baseline**

**6 weeks**

Supplement: Supplementary file 2 — Figure S1. Training-induced changes in the absolute counts of naive and memory phenotypes stratified for CMV. Note: Data are median values with error bars representing 95%CI. CMV = cytomegalovirus. *p < 0.05, increased significantly after exercise compared to baseline. Figure S2. The gating procedure and representative dot plots for the delineation of T-cell sub-populations by flow cytometry. Figure S2A (gating strategy), Figure S2B (representative plots for the strength endurance training group), Figure S2C (representative plots for the intensive strength training group) and Figure S2D (representative plots for the control group). (ZIP 625 kb) [file 12979_2019_157_MOESM2_ESM.zip › Supplementary Figure S2B R3.docx]

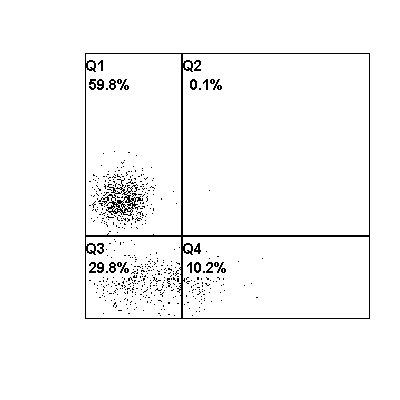

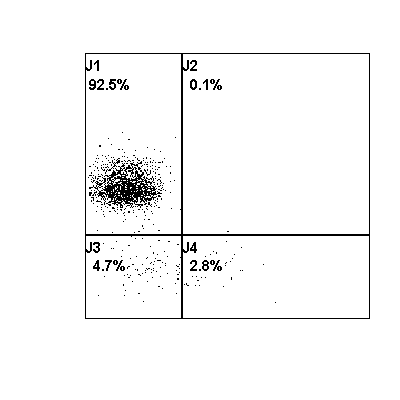

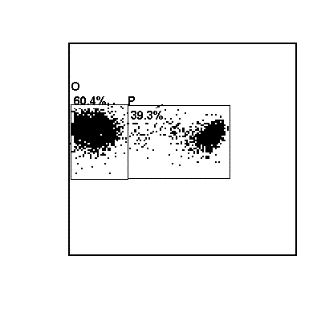

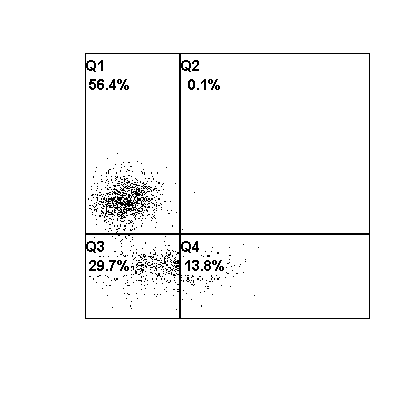

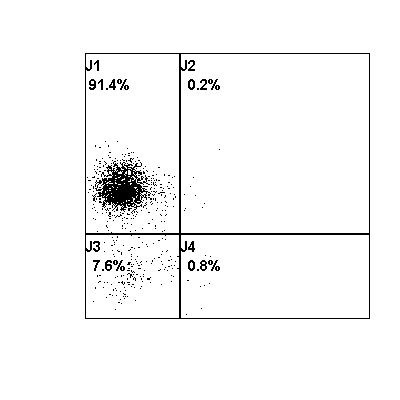

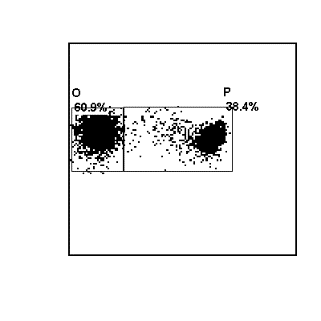


**CD3**

**CD8**

**CD57**

**CD57**

**CD28**

**CD28**

**CD8─**

**CD8+**

**CD3**

**CD8**

**CD57**

**CD57**

**CD28**

**CD28**

**CD8─**

**CD8+**

**Figure S2C**

**Baseline**

**6 weeks**

Supplement: Supplementary file 2 — Figure S1. Training-induced changes in the absolute counts of naive and memory phenotypes stratified for CMV. Note: Data are median values with error bars representing 95%CI. CMV = cytomegalovirus. *p < 0.05, increased significantly after exercise compared to baseline. Figure S2. The gating procedure and representative dot plots for the delineation of T-cell sub-populations by flow cytometry. Figure S2A (gating strategy), Figure S2B (representative plots for the strength endurance training group), Figure S2C (representative plots for the intensive strength training group) and Figure S2D (representative plots for the control group). (ZIP 625 kb) [file 12979_2019_157_MOESM2_ESM.zip › Supplementary Figure S2C R3.docx]

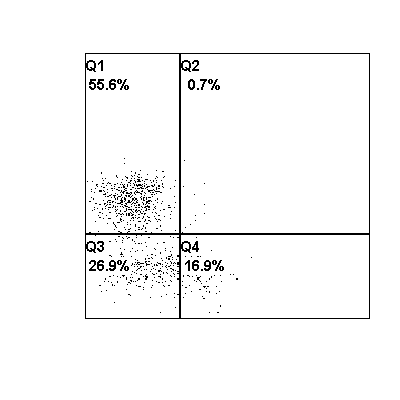

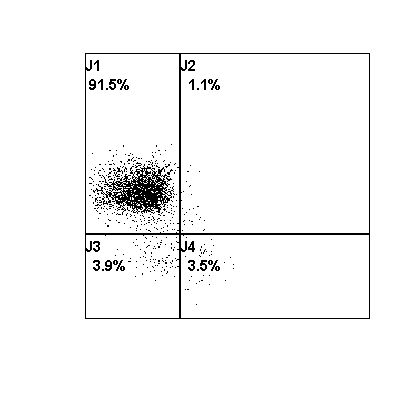

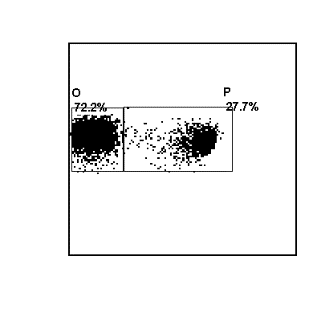

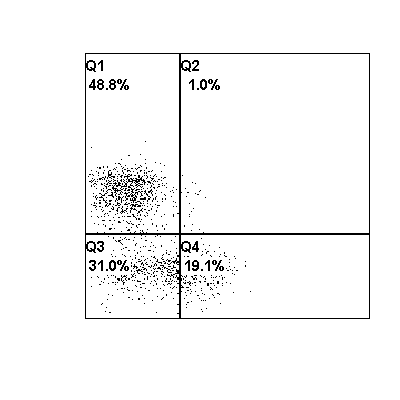

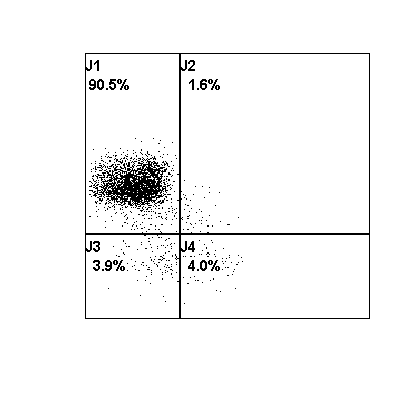

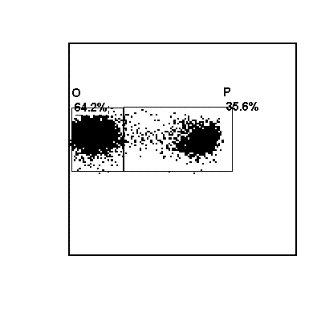


**CD3**

**CD8**

**CD57**

**CD57**

**CD28**

**CD28**

**CD8─**

**CD8+**

**CD3**

**CD8**

**CD57**

**CD57**

**CD28**

**CD28**

**CD8─**

**CD8+**

**Figure S2D**

**Baseline**

**6 weeks**

Supplement: Supplementary file 2 — Figure S1. Training-induced changes in the absolute counts of naive and memory phenotypes stratified for CMV. Note: Data are median values with error bars representing 95%CI. CMV = cytomegalovirus. *p < 0.05, increased significantly after exercise compared to baseline. Figure S2. The gating procedure and representative dot plots for the delineation of T-cell sub-populations by flow cytometry. Figure S2A (gating strategy), Figure S2B (representative plots for the strength endurance training group), Figure S2C (representative plots for the intensive strength training group) and Figure S2D (representative plots for the control group). (ZIP 625 kb) [file 12979_2019_157_MOESM2_ESM.zip › Supplementary Figure S2D R3.docx]
